# Supplementary material for: Experiences and actions related to living with type 1 diabetes during the COVID-19 pandemic in Norway: a qualitative study conducted during July to December 2020
Source: BMJ Open. 2022 Apr 7;12(4):e056027. doi: 10.1136/bmjopen-2021-056027 (PMC8990606; doi:10.1136/bmjopen-2021-056027)
Supplement: Author's manuscript [file bmjopen-2021-056027.draft_revisions.pdf]

BMJ Open is committed to open peer review. As part of this commitment we make the peer review history of every article we publish publicly available.

When an article is published we post the peer reviewers' comments and the authors' responses online. We also post the versions of the paper that were used during peer review. These are the versions that the peer review comments apply to.

The versions of the paper that follow are the versions that were submitted during the peer review process. They are not the versions of record or the final published versions. They should not be cited or distributed as the published version of this manuscript.

BMJ Open is an open access journal and the full, final, typeset and author-corrected version of record of the manuscript is available on our site with no access controls, subscription charges or pay-per-view fees (http://bmjopen.bmj.com).

If you have any questions on BMJ Open's open peer review process please email info.bmjopen@bmj.com

BMJ Open

Experiences and actions related to living with type 1 diabetes during the COVID-19 pandemic in Norway A critical incident technique analysis

Journal:	*BMJ Open*
Manuscript ID	bmjopen-2021-056027
Article Type:	Original research
Date Submitted by the Author:	03-Aug-2021
Complete List of Authors:	Pleym, Karin; Western Norway University of Applied Sciences, Department of Health and Caring Sciences; Vestre Viken Hospital Trust, Department of Medicine<br>Iversen, Marjolein; Western Norway University of Applied Sciences, Department of Health and Caring Sciences<br>Broström, Anders; Jönköping University, Department of Nursing, School of Health and Welfare; Linköping University Hospital, Department of Clinical Neurophysiology
Keywords:	COVID-19, QUALITATIVE RESEARCH, DIABETES & ENDOCRINOLOGY

SCHOLARONE™
Manuscripts

I, the Submitting Author has the right to grant and does grant on behalf of all authors of the Work (as defined in the below author licence), an exclusive licence and/or a non-exclusive licence for contributions from authors who are: i) UK Crown employees; ii) where BMJ has agreed a CC-BY licence shall apply, and/or iii) in accordance with the terms applicable for US Federal Government officers or employees acting as part of their official duties; on a worldwide, perpetual, irrevocable, royalty-free basis to BMJ Publishing Group Ltd ("BMJ") its licensees and where the relevant Journal is co-owned by BMJ to the co-owners of the Journal, to publish the Work in this journal and any other BMJ products and to exploit all rights, as set out in our licence.

The Submitting Author accepts and understands that any supply made under these terms is made by BMJ to the Submitting Author unless you are acting as an employee on behalf of your employer or a postgraduate student of an affiliated institution which is paying any applicable article publishing charge ("APC") for Open Access articles. Where the Submitting Author wishes to make the Work available on an Open Access basis (and intends to pay the relevant APC), the terms of reuse of such Open Access shall be governed by a Creative Commons licence – details of these licences and which Creative Commons licence will apply to this Work are set out in our licence referred to above.

Other than as permitted in any relevant BMJ Author's Self Archiving Policies, I confirm this Work has not been accepted for publication elsewhere, is not being considered for publication elsewhere and does not duplicate material already published. I confirm all authors consent to publication of this Work and authorise the granting of this licence.

For peer review only

Experiences and actions related to living with type 1 diabetes during the COVID-19 pandemic in Norway

A critical incident technique analysis

Karin Pleym RN[1,2], Marjolein M. Iversen[1] and Anders Broström[3,4]

1. Faculty of Health and Social Science, Western University of Applied Sciences, Norway

2. Department of Medicine, Vestre Viken Drammen Hospital, Norway

3. Department of Nursing, School of Health and Welfare, Jönköping University, Jönköping, Sweden

4. Department of Clinical Neurophysiology, Linköping University Hospital, Linköping, Sweden

Corresponding author: Marjolein M. Iversen, Inndalsveien 28, 5063 Bergen, Norway. Tel. +47 902 55 044, e-mail: Marjolein.Memelink.Iversen@hvl.no

Word count:

Abstract: 293 words

Manuscript: 3999

Tables: 4

Figures: 0

References: 48

For peer review only

Abstract

Introduction: Diabetes has been identified as one of several underlying health conditions associated with increased risk of severe illness and death from SARS-CoV-2 virus infection (COVID-19). During the COVID-19 pandemic, patients with comorbidities such as diabetes were advised to follow strict pandemic guidance on infection control measures. The aim of this study is to describe the experiences of adults with type 1 diabetes (T1DM) during the COVID-19 pandemic in Norway, and what actions they took to cope with the situation.

Research design and methods: An inductive, descriptive design applying the critical incident technique (CIT) was used. A strategic selection was made from diabetes specialist outpatient clinics at three different hospitals in eastern Norway. Descriptions of incidents and actions were obtained through semi-structured individual interviews with 19 people with T1DM.

Results: Experiences were categorized into two main areas: "Increased psychosocial burden of T1DM during the COVID-19 pandemic" and "Changed conditions for T1DM treatment during the COVID-19 pandemic". Uncertainty distress and social consequences from infection control measures contributed to the burden of T1DM. Disrupted T1DM follow-up and altered daily routines created challenges. However, having more time to focus on T1DM self-management during lockdown represented an improvement. Actions to adapt to the situation included strategies such as avoiding media coverage of COVID-19, stocking up on insulin, actively searching for personalized information and positive thinking. Actions were categorized into two main areas: "Actions to handle psychosocial strain related to T1DM and COVID-19" and "Actions to handle changed conditions for T1DM treatment during the COVID-19 pandemic".

Conclusions: During the COVID-19 pandemic patients experienced an increased psychosocial burden of T1DM and difficulties from a disrupted daily life affecting T1DM self-management routines. Uncertainty-reducing behaviours and actions to adapt to the situation provided a general sense of coping despite difficulties.

KEYWORDS: COVID-19; diabetes; psychosocial health; qualitative research

For peer review only

Strengths and limitations of this study

- To our knowledge, this paper provides the first evidence of the experiences and actions of people with T1DM during the COVID-19 pandemic in Norway
- The informants were recruited from diabetes specialist outpatient clinics situated in both rural and urban areas
- Out of 19 interviews, 14 were conducted by video call and three were conducted by telephone
- We aimed to achieve variation in gender, age, metabolic control, T1DM duration and occupation
- A diversity of experiences and actions were obtained from the interviews

For peer review only

INTRODUCTION

The outbreak of SARS-CoV-2-induced coronavirus disease 2019 (COVID-19) was declared a global pandemic by the World Health Organization on 11 March 2020.[1] The COVID-19 disease ranges in severity from asymptomatic or mild cases to severe illness and death.[2, 3] Diabetes has been identified as one of several underlying health conditions associated with increased severity and higher mortality rates from COVID-19 illness.[4–9]

In Norway the government implemented strict interventions to limit the spread of the virus.[10] The infection control measures had significant impacts on the population's everyday life. Previous reports from preceding coronavirus epidemics such as SARS-CoV (SARS) in 2002–2003 and the Middle East Respiratory Syndrome coronavirus (MERS) in 2012–2015 showed evidence of increased psychosocial and mental health challenges.[11–13]

Similarly, psychosocial challenges caused by the COVID-19 pandemic have been seen in the general population.[14–16] The massive media coverage and the general perception of the severity of the situation had potential to cause stress and anxiety. People with type 1 diabetes (T1DM) can be more vulnerable to stress in terms of a direct effect of stress hormones on blood glucose levels and an indirect effect on the person’s behaviour related to diabetes self-management.[17] Previous studies have shown that people with T1DM experienced disrupted follow-up from specialist outpatient clinics during the COVID-19 pandemic and challenges affecting T1DM self-management routines.[18–22] Other studies, however, showed improved glycaemic control in periods of lockdown of society.[23–25] A cross-sectional survey from Denmark, in which 2 430 adults with diabetes completed an online questionnaire, indicated that people with diabetes have COVID-19-specific worries about their diabetes associated with poorer psychosocial health.[26] In line with these results, diabetes nurses across Europe have reported a significant increase in physical and psychological problems in patients with diabetes.[27] A qualitative study has provided insight into the experiences of people diagnosed with various types of diabetes in the initial phase of lockdown in Denmark.[28] However, little is known about how people with T1DM handled daily life during the pandemic over a longer period of time that includes the reopening of society. Knowledge of the experiences and actions of people with T1DM during

For peer review only

the COVID-19 pandemic may provide insight into strategies for handling the situation as well as possible needs for assistance from the healthcare system. This knowledge may in turn be valuable for other risk groups and in the event of similar situations in future. The aim of this study was to describe the experiences of adults with T1DM during the COVID-19 pandemic in Norway and what actions they took to cope with their situation.

RESEARCH DESIGN AND METHODS

Design and method description

This qualitative study used an inductive, descriptive design, applying the critical incident technique (CIT).[29] A "critical incident" represents a major event of great importance to the person involved, and is an essential part of the CIT methodology. Critical incidents consist of positive or negative experiences in addition to actions carried out to handle the phenomenon in question.[30] Due to its flexibility and the focus on the patient perspective, CIT is considered an eligible method for use in healthcare research.[31] The method enables informants to describe significant incidents as well as their experiences and actions. Consequently, CIT is a suitable method for exploring the research question of this study.

The study protocol was evaluated by the Regional Committe for Medical and Health Research Ethics in South East Norway (REC-154546), the Norwegian Centre for Research Data (NSD) (NSD-39284), the local data protection officers at the three study sites and Western Norway University of Applied Science before study start.

Patient and Public Involvement statement

The study had a patient advisor from the local diabetes association who reviewed the interview guide and gave advice on the content. Data are patient reported and results are based on interviews. Feedback regarding the results is planned through local and national meetings/journals.

For peer review only

Participants and setting

The informants were recruited from the diabetes specialist outpatient clinics at three different hospitals in eastern Norway. The hospitals, two community hospitals and one university hospital, were situated in both rural and urban areas. The total patient populations at the hospitals were 160 000, 240 000 and 560 000 respectively. People with T1DM will typically meet with a diabetes nurse or an endocrinologist in the diabetes outpatient clinics every three to six months.

A strategic selection was made to ensure a heterogeneous group of informants. Inclusion criteria were people with T1DM aged 18–65 years. Exclusion criteria were pregnancy, people with chronic pulmonary disorders, people with active cancer diseases and people diagnosed with a myocardial infarction or stroke during the previous six months. Characteristics such as gender, age, T1DM duration, metabolic control and having an occupation with increased exposure to possible infection were used in the selection of informants (Table 1). Based on the criteria for strategic selection, a local designated diabetes nurse provided oral information about the study to potential participants. When interest was expressed, the nurse asked for oral consent to be contacted by the first author by telephone to provide further information about the study. The first author then made contact by telephone and gave additional information, and written information was sent to the potential participant by mail. After written informed consent was returned to the first author, an appointment for an interview was made. Three informants later cancelled the interview appointment due to a busy schedule (2) or illness (1). In total, 19 people with T1DM were included in the study.

-Insert Table 1

Data collection

Descriptions of experiences and actions were obtained through semi-structured individual interviews. Due to the ongoing pandemic, informants could choose to conduct the interviews in person, by video call or by telephone. The video interviews were conducted on an encrypted web platform.

A semi-structured interview was performed based on an interview guide. The guide was established based on literature, clinical experience, and thorough discussion in the research

For peer review only

team. After three interviews were conducted, the transcriptions were discussed within the research team. The interview guide consisted of three main topics and probing questions as presented in Table 2.

The interviews were all performed by the first author and recorded on a digital recorder. The first author transcribed 12 and a project assistant transcribed seven of the audio recordings verbatim. The interviews were performed between July 2020 and December 2020. A total of 313 experiences and 185 actions was identified and categorized.

-Insert Table 2

Data analysis

The study was analysed in line with CIT as described by Fridlund et al.[30] The first author read the interviews several times to obtain an overall picture of the content. Experiences and actions related to the purpose of the study were highlighted in the text and delimited. Delimitations from the text were then compared for similarities in an effort to group them into subcategories. When the subcategories were identified, a new process of exploration and comparison was performed to group the subcategories into categories. The purpose of a category is to describe the general character of the subcategories. Following the establishment of categories, a new effort was made to group categories into main areas. The purpose of constructing main areas was to describe the overall theme of the data. Descriptions of experiences and actions were analysed separately. This process led to the finding of 21 subcategories of experiences and 18 subcategories of actions. The subcategories were then structured into six categories of experiences and five categories of actions. Finally, two main areas of experiences and actions were identified. Categorization was regularly discussed in the research team until agreement was achieved.

RESULTS

In total, 10 female and nine male people with T1DM participated in the study, of which two had been diagnosed with COVID-19. All the informants were Norwegian born with the same cultural background (Table 1).

For peer review only

Experiences

Table 3 presents the experiences of people with T1DM during the COVID-19 pandemic in Norway, including direct quotations from the interviews. Two main areas emerged from the data analysis of experiences: "Increased psychosocial burden of T1DM during the COVID-19 pandemic" and "Changed conditions for T1DM treatment during the COVID-19 pandemic".

-Insert Table 3

Increased psychosocial burden of T1DM during the COVID-19 pandemic

Concerns related to COVID-19 and T1DM

Informants described uncertainty caused by belonging to a risk group. This included fear of getting sick from COVID-19 and confusion about the reasons why T1DM was associated with higher risk of a fatal outcome. General anxiety about the uncertainty of the overall situation was described as difficult.

A need for knowledge about T1DM and COVID-19

The continuous media coverage caused anxiety and worries. The informants expressed a need for balanced information aimed at people in risk groups. Additionally, personalized information was desired and sought by contacting familiar healthcare providers such as the general practitioner or the diabetes nurse.

Consequences for social relations as a result of COVID-19-related restrictions

This category describes the various difficulties the informants experienced while socializing with family, friends, colleagues and others. To avoid bringing the COVID-19 virus into the home, family members of people with T1DM were subjected to stricter restrictions than their peers. For instance, the children of one informant were not allowed to use public transport, and the son of a divorced woman moved temporarily to live with his father.

For peer review only

Experiences of social limitations in the workplace due to the T1DM diagnosis were reported. For example, one of the informants was told that it was not desirable for her to return to the workplace after the termination of lockdown, as colleagues were afraid of infecting her.

Changed conditions for T1DM treatment during the COVID-19 pandemic

Altered routines due to COVID-19 affecting T1DM self-management

Restrictions implemented by the government to prevent the spread of COVID-19 created changes in daily routines which in turn affected T1DM self-management. Informants expressed difficulties in maintaining a healthy lifestyle in terms of physical activity and healthy diet. To have a good time within the family, food such as cakes and ice cream were served more frequently. Since the gyms were closed, less familiar activities were initiated which made it more difficult to predict correct insulin doses. Informants who contracted COVID-19 experienced large fluctuations in and high levels of blood glucose during the period of illness. Having access to continuous blood glucose monitoring (CGM) and insulin pumps was seen as an advantage in handling this situation. Reaching out to the diabetes nurse by telephone for advice on how to titrate insulin doses gave a sense of security.

Changes in the work or study situation due to COVID-19 affecting T1DM self-management

Measures to prevent infection in the workplace led to new routines. This had an impact on meal breaks, time spent being physically active and opportunities for social contact at work. Some, such as a teacher and a mail delivery worker, experienced increased work pressure and new work tasks, whereas others experienced a more predictable daily life with less stress and more time to take care of T1DM self-management. Being laid off from work because of lockdown gave rise to financial insecurity. This led to stress which in turn affected blood glucose levels.

Reorganization of the health service due to COVID-19 affecting T1DM-treatment

For peer review only

Informants experienced delayed follow-up from the specialist outpatient clinic. This was due to the reorganization of hospital resources to prepare for a large number of COVID-19 patients. The authorities introduced a temporary rationing of insulin to prevent hoarding and ensure access. This led to concerns about the availability of medications and created worries about the inconvenience of having to go to the pharmacy more frequently. Informants' concerns about possible lack of competence in the intensive care unit regarding specialized T1DM treatment made the risk of becoming severely ill from COVID-19 even more frightening. These concerns related to previous experiences from hospitalizations. Having experienced easy access to healthcare professionals before the pandemic made them feel confident that they could reconnect for information and advice when needed.

Actions

Table 4 presents the actions of people with T1DM during the COVID-19 pandemic in Norway, including direct quotations from the interviews. The two main areas of actions that emerged from the data analysis were "Actions to handle psychosocial strain related to T1DM and COVID-19" and "Actions to handle changed conditions for T1DM treatment during the COVID-19 pandemic". The analysis of actions is described in Table 4.

-Insert Table 4

Actions to handle psychosocial strain related to T1DM and COVID-19

Practical strategies to avoid being infected with COVID-19

Informants described increased awareness of complying with general advice on infection control. Measures such as social distancing and frequent hand-washing became a daily routine. Some started using a mask early on. Avoiding crowded places and receiving help from others for grocery shopping and providing necessary medications were strategies adopted to prevent infection. Limiting the number of social contacts for the family as a whole was considered necessary to avoid infection.

For peer review only

Measures to maintain social contact

Informants described finding support and joy within the family. Choosing a few people to meet regularly gave them the opportunity to maintain social contact outside the home. Children of parents with T1DM were allowed to choose one or two friends to play with. Both new and familiar digital platforms served as alternative arenas for social interaction.

Mental strategies to deal with the situation

Informants sought personalized information about their risk status. Positive thinking was one of the main strategies to cope with the overall situation. Furthermore, informants actively avoided media coverage of COVID-19.

Actions to handle changed conditions for T1DM treatment during the COVID-19 pandemic

Creating a safe structure in daily life

Predictable routines were created to facilitate T1DM self-management. New job routines were implemented to protect the informants from being infected. Working from home was challenging, but establishing home-office routines was experienced as a positive factor. Lack of routine made handling work and T1DM self-management difficult. Meaningful activities at home were found helpful to avoid pondering about the situation.

Measures to ensure proper T1DM self- management during the COVID-19 pandemic

Medical devices such as CGM and insulin pumps were perceived as useful in the treatment of T1DM and contributed to good metabolic control. Furthermore, informants found alternative physical activities to maintain a healthy lifestyle when the fitness centres were closed. Keeping a storage of insulin led to a sense of security. For those who contracted COVID-19, high blood glucose levels caused by the infection were met with efforts to titrate insulin doses, and informants called the diabetes nurse for support and advice on this matter.

For peer review only

DISCUSSION

This study aimed to describe the experiences and actions of people with T1DM during the COVID-19 pandemic in Norway. Results indicate that the psychosocial burden of living with T1DM increased during the pandemic. Uncertainty caused by belonging to a risk group, fear of severe illness and strict adherence to social distancing for the family as a whole contributed to the burden of T1DM. Additionally, we found that infection control measures and reorganization of the healthcare system represented challenges in the form of changed conditions for T1DM treatment. Applying manageable, understandable and meaningful actions to deal with the psychosocial strain and the disrupted daily life led to a general sense of coping despite difficulties.

Alteration of daily routines brought challenges such as an increased amount of unhealthy food and less exercise. These findings are in line with previous quantitative research.[32, 33] In a Saudi cross-sectional study of people with T1DM using an insulin pump, difficulties in maintaining a healthy diet and staying physically active during lockdown were seen. However, adherence to the insulin pump therapy contributed to stabilizing blood glucose.[32] Participants in our study described that general stress and anxiety affected T1DM self-management in terms of fluctuations in blood glucose similar to those seen in studies from Italy.[22, 34] Nevertheless, our participants also described a more predictable lifestyle in working from home that led to improved control of blood glucose levels. Similar findings of improved metabolic control have been reported from studies of children with T1DM on insulin pump therapy.[35, 36] Furthermore, multiple studies that reviewed metabolic control via CGM data registered during the pandemic found improvements in both time in range and blood glucose levels,[23–25] although one study showed a tendency of increased fluctuations in physical activity and perceived stress.[34]

The finding of an increased psychosocial burden of T1DM during the COVID-19 pandemic is consistent with existing evidence from quantitative studies.[26, 27] Our study provides an in-depth understanding of these psychosocial difficulties, and is consistent with a qualitative study conducted in Denmark.[28] While the Danish study focused on the experiences of

For peer review only

people with multiple types of diabetes during lockdown in Denmark (April 2020), the results from our study focused on people with T1DM at a later stage of the pandemic (July 2020–December 2020). Therefore, we covered experiences related to the reopening of society as well as to new periods of lockdown. In comparison with the Danish study, our findings may indicate that the psychosocial burden of T1DM during the pandemic increased over time. This interpretation is supported by another qualitative study of people living with different long-term physical health conditions during the COVID-19 pandemic. In this study they found that the COVID-19 pandemic affected several aspects of mental health and well-being.[16] Furthermore, in a review of the effects of quarantine, impacts on psychosocial health were seen, and the length of the period of isolation affected the outcomes of these negative effects.[12] Even though our participants were not quarantined, they were to some extent self-isolating for extended periods, and thus the psychosocial burden might have increased accordingly.

"Diabetes distress" refers to a psychological condition associated with the burden of living with T1DM.[37] Diabetes distress might add to general stress from the severity of the pandemic situation and further increase the psychosocial burden of T1DM. In a previous study, diabetes distress was identified as one of several factors associated with poorer psychosocial health in patients with diabetes during the COVID-19 pandemic.[26]

Freeston et al.[38] have proposed a model of uncertainty distress in the context of the COVID-19 pandemic. According to this model, uncertainty can be defined as “the subjective negative emotions experienced in response to the as yet unknown aspects of a given situation.”[38] The degree of uncertainty distress depends on the following factors: disruption of everyday life, perceived actual threat and perceived uncertainty. In addition, individual tolerance of uncertainty influences uncertainty distress. Individual tolerance of uncertainty can be modified by uncertainty-reducing behaviours. Such behaviours may include actions such as avoidance, preparation for possible negative consequences, postponement and the search for reassurance. The actions taken by the participants in our study can be seen in the light of this model of uncertainty distress in the context of the COVID-19 pandemic. Our participants actively avoided media, they prepared for possible negative consequences by keeping a stock of insulin and by strictly complying with advices

For peer review only

on infection control measures. Furthermore, social gatherings were postponed, and the participants searched for tailored and personalized information for reassurance.

In this study, we have described how various actions were taken by the participants to adapt, and positive thinking was one of the main strategies to cope with the overall situation. From the perspective of the theory of salutogenesis, stressors in life will not necessarily contribute to poor outcomes of health if met properly. The essential factor is that the individual finds understandable, manageable and meaningful actions to adapt. Through meaningful actions, people may achieve a sense of coherence and thus of coping despite challenging environments.[39–41]

Participants in our study emphasized the importance of access to healthcare professionals by telephone for personalized guidance. A recently published but not yet peer-reviewed study from Norway shows a surprising and significant increase in non-COVID-related deaths in patients with diabetes from March 2020 to May 2020.[42] The causal factors behind these findings are unknown, and it is not possible to draw conclusions. Nevertheless, Raknes et al. propose inappropriate avoidance of healthcare professionals and thus a lack of follow-up as a possible explanation. Based on this understanding, the importance of having healthcare professionals such as diabetes nurses available by telephone for medical advice and social support is further underlined.

Study limitations

We have strived to achieve trustworthiness throughout the study in terms of credibility, dependability, confirmability and transferability as described by Polit & Beck.[43] Several researchers with good knowledge of both CIT and diabetes have been involved throughout the study. The first author (diabetes nurse specialist) has clinical experience which includes answering calls from diabetes patients anxious about COVID-19. Her experience represents a factor that might threaten reflexivity. On the other hand, substantial knowledge from the field can be a valuable source of relevant and specific research.[44] The first author's clinical experience can be seen as an advantage in the process of formulating relevant questions for the interview guide. Awareness of the potential influence of previous experiences has been taken into account in the interpretation

For peer review only

of the data. Description of the data has sought to be clear and has been discussed in depth within the research team to strengthen the credibility of the study. The interviews were all performed by the first author, which increased the likelihood that the interview guide was used in the same way in all the interviews. Out of 19 interviews, 14 were conducted by video call and three were conducted by telephone, which may have contributed to a loss of some nonverbal data.[45] On the other hand, participants had the comfort of familiar environments and did not have to worry about possible infection. In sampling, we aimed to achieve variation in gender, age, metabolic control, T1DM duration and occupation. However, we were unable to include participants with a different cultural background, which is a limitation. To strengthen dependability and facilitate confirmability, the systematic procedure we followed in the data analysis is clearly presented with quotations categorised and finally grouped into main areas. A diversity of experiences and actions were obtained from the interviews, and this is seen as a strength when using the CIT approach.[29]

According to Malterud, the field and the context in which the data material is acquired will always determine the scope of the knowledge in time and space.[46] From an international perspective, Norway has had relatively few deaths from COVID-19 compared to other European countries.[47] As of the 28 April 2021 there are 753 registered deaths out of 111 685 confirmed COVID-19 cases in a population of 5 391 369 inhabitants.[48] Participants included in our study live in the eastern part of Norway close to the capital. The city of Oslo and surrounding areas are the part of Norway hardest affected by the COVID-19 pandemic. From this perspective, it would be fair to say that the participants in this study may have had experiences similar to people in countries with higher infection rates and deaths from COVID-19. A detailed description of the study context, the participants, method of data collection and the data analysis, including direct quotations from the interviews, are presented (Table 1, Table 2, Table 3 and Table 4) to give the reader the opportunity to determine the transferability of the findings.

Conclusion and implications for clinical practice

In conclusion, participants experienced psychosocial strain and an increased burden of T1DM. Furthermore, they described uncertainty-reducing behaviours and actions which

provided a general sense of coping with handling a disrupted daily life during the COVID-19 pandemic despite difficulties.

Healthcare professionals should be aware that many people with T1DM might have lived under increased stress during the COVID-19 pandemic. Availability of healthcare professionals for personalized advice by telephone was emphasized as important to alleviate uncertainty distress, reduce diabetes distress and support adequate T1DM self-management. Access to telephone and video consultations should be implemented to ensure follow-up of people with T1DM in similar situations in future.

Further research is needed to investigate potential psychosocial impacts of the COVID-19 pandemic on people with T1DM from a family perspective. Exploring possible associations between diabetes distress, uncertainty distress and psychosocial burden was beyond the scope of this study. However, this would be an interesting topic for future research.

Competing interests: None

Funding: Karin Pleym has applied for a contribution of NOK 10 000 from the regional Diabetes Association for the project.

Contributorship statement: KP contributed to the study concept, project planning and management, recruitment of participants, data analysis, manuscript preparation, drafting the manuscript and approved the final manuscript. AB contributed to the study concept, project planning, data analysis, manuscript preparation, drafting the manuscript and approved the final manuscript. MMI contributed to the study concept, project planning and management, data analysis, manuscript preparation, drafting the manuscript and approved the final manuscript.

Data sharing statement: No additional data available.

For peer review only

References

1 WHO. WHO Director-General’s opening remarks at the media briefing on COVID-19 - 11 March 2020. 2020.https://www.who.int/director-general/speeches/detail/who-director-general-s-opening-remarks-at-the-media-briefing-on-covid-19---11-march-2020 (accessed 10 Mar 2021).

2 Chen G, Wu D, Guo W, *et al.* Clinical and immunological features of severe and moderate coronavirus disease 2019. *J Clin Invest* 2020;**130**:2620–9. doi:10.1172/JCI137244

3 Zhou F, Yu T, Du R, *et al.* Clinical course and risk factors for mortality of adult inpatients with COVID-19 in Wuhan, China: a retrospective cohort study. *The Lancet* 2020;**395**:1054–62. doi:10.1016/S0140-6736(20)30566-3

4 Zhu L, She Z-G, Cheng X, *et al.* Association of Blood Glucose Control and Outcomes in Patients with COVID-19 and Pre-existing Type 2 Diabetes. *Cell Metab* 2020;**31**:1068-1077.e3. doi:10.1016/j.cmet.2020.04.021

5 McGurnaghan SJ, Weir A, Bishop J, *et al.* Risks of and risk factors for COVID-19 disease in people with diabetes: a cohort study of the total population of Scotland. *Lancet Diabetes Endocrinol* 2021;**9**:82–93. doi:10.1016/S2213-8587(20)30405-8

6 Barron E, Bakhai C, Kar P, *et al.* Associations of type 1 and type 2 diabetes with COVID-19-related mortality in England: a whole-population study. *Lancet Diabetes Endocrinol* 2020;**8**:813–22. doi:10.1016/S2213-8587(20)30272-2

7 Xu L, Mao Y, Chen G. Risk factors for 2019 novel coronavirus disease (COVID-19) patients progressing to critical illness: a systematic review and meta-analysis. *Aging* 2020;**12**:12410–21. doi:10.18632/aging.103383

8 Huang C, Wang Y, Li X, *et al.* Clinical features of patients infected with 2019 novel coronavirus in Wuhan, China. *The Lancet* 2020;**395**:497–506. doi:10.1016/S0140-6736(20)30183-5

9 Kumar A, Arora A, Sharma P, *et al.* Is diabetes mellitus associated with mortality and severity of COVID-19? A meta-analysis. *Diabetes Metab Syndr Clin Res Rev* 2020;**14**:535–45. doi:10.1016/j.dsx.2020.04.044

10 Regjeringen. Omfattende tiltak for å bekjempe koronaviruset. 2020.https://www.regjeringen.no/no/aktuelt/nye-tiltak/id2693327/ (accessed 10 Nov 2020).

11 Blendon RJ, Benson JM, DesRoches CM, *et al.* The Public’s Response to Severe Acute Respiratory Syndrome in Toronto and the United States. *Clin Infect Dis* 2004;**38**:925–31. doi:10.1086/382355

12 Brooks SK, Webster RK, Smith LE, *et al.* The psychological impact of quarantine and how to reduce it: rapid review of the evidence. *The Lancet* 2020;**395**:912–20. doi:10.1016/S0140-6736(20)30460-8

13 Yoon M-K, Kim S-Y, Ko H-S, *et al.* System effectiveness of detection, brief intervention and refer to treatment for the people with post-traumatic emotional distress by MERS: a case report of community-based proactive intervention in South Korea. *Int J Ment Health Syst* 2016;**10**:51. doi:10.1186/s13033-016-0083-5

For peer review only

14 Hetkamp M. Sleep disturbances, fear, and generalized anxiety during the COVID-19 shut down phase in Germany: relation to infection rates, deaths, and German stock index DAX. *Sleep Med* 2020;:4.

15 Yu H, Li M, Li Z, *et al.* Coping style, social support and psychological distress in the general Chinese population in the early stages of the COVID-19 epidemic. *BMC Psychiatry* 2020;**20**:426. doi:10.1186/s12888-020-02826-3

16 Fisher A, Roberts A, McKinlay AR, *et al.* The impact of the COVID-19 pandemic on mental health and well-being of people living with a long-term physical health condition: a qualitative study. Public and Global Health 2020. doi:10.1101/2020.12.03.20243246 [PREPRINT]

17 Marcovecchio ML, Chiarelli F. The effects of acute and chronic stress on diabetes control. *Sci Signal* 2012;**5**:pt10. doi:10.1126/scisignal.2003508

18 Ikesu R, Miyawaki A, Sugiyama T, *et al.* Trends in Diabetes Care during the COVID-19 Outbreak in Japan: an Observational Study. *J Gen Intern Med* Published Online First: 19 January 2021. doi:10.1007/s11606-020-06413-w

19 Pal R, Yadav U, Verma A, *et al.* Awareness regarding COVID-19 and problems being faced by young adults with type 1 diabetes mellitus amid nationwide lockdown in India: A qualitative interview study. *Prim Care Diabetes* 2021;**15**:10–5. doi:10.1016/j.pcd.2020.07.001

20 Shi C, Zhu H, Liu J, *et al.* Barriers to Self-Management of Type 2 Diabetes During COVID-19 Medical Isolation: A Qualitative Study. *Diabetes Metab Syndr Obes Targets Ther* 2020;**Volume 13**:3713–25. doi:10.2147/DMSO.S268481

21 Verma A, Rajput R, Verma S, *et al.* Impact of lockdown in COVID 19 on glycemic control in patients with type 1 Diabetes Mellitus. *Diabetes Metab Syndr Clin Res Rev* 2020;**14**:1213–6. doi:10.1016/j.dsx.2020.07.016

22 Barchetta I, Cimini FA, Bertoccini L, *et al.* Effects of work status changes and perceived stress on glycaemic control in individuals with type 1 diabetes during COVID-19 lockdown in Italy. *Diabetes Res Clin Pract* 2020;**170**:108513. doi:10.1016/j.diabres.2020.108513

23 Aragona M, Rodia C, Bertolotto A, *et al.* Type 1 diabetes and COVID-19: The "lockdown effect." *Diabetes Res Clin Pract* 2020;**170**. doi:10.1016/j.diabres.2020.108468

24 Potier L, Hansel B, Larger E, *et al.* Stay-at-Home Orders During the COVID-19 Pandemic, an Opportunity to Improve Glucose Control Through Behavioral Changes in Type 1 Diabetes. *Diabetes Care* 2021;**44**:839–43. doi:10.2337/dc20-2019

25 Fernández E, Cortazar A, Bellido V. Impact of COVID-19 lockdown on glycemic control in patients with type 1 diabetes. *Diabetes Res Clin Pract* 2020;**166**:108348. doi:10.1016/j.diabres.2020.108348

26 Joensen LE, Madsen KP, Holm L, *et al.* Diabetes and COVID-19: psychosocial consequences of the COVID-19 pandemic in people with diabetes in Denmark—what characterizes people with high levels of COVID-19-related worries? *Diabet Med* 2020;**37**:1146–54. doi:10.1111/dme.14319

27 Forde R, Arente L, Ausili D, *et al.* The impact of the COVID-19 pandemic on people with diabetes and diabetes services: A pan-European survey of diabetes specialist nurses undertaken by the

For peer review only

Foundation of European Nurses in Diabetes survey consortium. *Diabet Med* Published Online First: 30 December 2020. doi:10.1111/dme.14498

28 Grabowski D, Overgaard M, Meldgaard J, *et al.* Disrupted Self-Management and Adaption to New Diabetes Routines: A Qualitative Study of How People with Diabetes Managed Their Illness during the COVID-19 Lockdown. *Diabetology* 2021;**2**:1–15. doi:10.3390/diabetology2010001

29 Flanagan JC. The critical incident technique. *Psychol Bull* 1954;**51**:327–58. doi:10.1037/h0061470

30 Fridlund B, Henricson, M., Mårtensson J. Critical Incident Technique applied in nursing and healthcare sciences. *SOJ Nurs Health Care* 2017;**3**:1–5. doi:10.15226/2471-6529/3/1/00125

31 Kemppainen JK. The critical incident technique and nursing care quality research. *J Adv Nurs* 2000;**32**:1264–71. doi:10.1046/j.1365-2648.2000.01597.x

32 Magliah SF, Zarif HA, Althubaiti A, *et al.* Managing Type 1 Diabetes among Saudi adults on insulin pump therapy during the COVID-19 lockdown. *Diabetes Metab Syndr Clin Res Rev* 2021;**15**:63–8. doi:10.1016/j.dsx.2020.12.013

33 Barone MTU, Harnik SB, de Luca PV, *et al.* The impact of COVID-19 on people with diabetes in Brazil. *Diabetes Res Clin Pract* 2020;**166**:108304. doi:10.1016/j.diabres.2020.108304

34 Di Dalmazi G, Maltoni G, Bongiorno C, *et al.* Comparison of the effects of lockdown due to COVID-19 on glucose patterns among children, adolescents, and adults with type 1 diabetes: CGM study. *BMJ Open Diabetes Res Care* 2020;**8**:e001664. doi:10.1136/bmjdrc-2020-001664

35 Tornese G, Ceconi V, Monasta L, *et al.* Glycemic Control in Type 1 Diabetes Mellitus During COVID-19 Quarantine and the Role of In-Home Physical Activity. *Diabetes Technol Ther* 2020;**22**:462–7. doi:10.1089/dia.2020.0169

36 Christoforidis A, Kavoura E, Nemtsa A, *et al.* Coronavirus lockdown effect on type 1 diabetes management on children wearing insulin pump equipped with continuous glucose monitoring system. *Diabetes Res Clin Pract* 2020;**166**:108307. doi:10.1016/j.diabres.2020.108307

37 Fisher L, Hessler D, Polonsky W, *et al.* Diabetes distress in adults with type 1 diabetes: Prevalence, incidence and change over time. *J Diabetes Complications* 2016;**30**:1123–8. doi:10.1016/j.jdiacomp.2016.03.032

38 Freeston M, Tiplady A, Mawn L, *et al.* Towards a model of uncertainty distress in the context of Coronavirus (COVID-19). *Cogn Behav Ther* 2020;**13**:e31. doi:10.1017/S1754470X2000029X

39 Lindstrom B. Contextualizing salutogenesis and Antonovsky in public health development. *Health Promot Int* 2006;**21**:238–44. doi:10.1093/heapro/dal016

40 Antonovsky A, Sjøbu A. *Helsens mysterium: den salutogene modellen*. Oslo: : Gyldendal akademisk 2013.

41 Bauer GF, Eriksson M, Espnes GA, *et al.*, editors. *The Handbook of Salutogenesis*. 1st ed. 2017. Cham: : Springer International Publishing : Imprint: Springer 2017. doi:10.1007/978-3-319-04600-6

For peer review only

42 Raknes G, Strøm MS, Sulo G, *et al.* Lockdown and non-COVID-19 deaths: Cause-specific mortality during the first wave of the 2020 pandemic in Norway. A population-based register study. Epidemiology 2021. doi:10.1101/2021.02.09.21251326 [PREPRINT]

43 Polit DF, Beck CT. *Nursing research: generating and assessing evidence for nursing practice*. Tenth edition. Philadelphia: : Wolters Kluwer Health 2017.

44 Malterud K. Qualitative research: standards, challenges, and guidelines. *The Lancet* 2001;**358**:483–8. doi:10.1016/S0140-6736(01)05627-6

45 Knox S, Burkard AW. Qualitative research interviews. *Psychother Res* 2009;**19**:566–75. doi:10.1080/10503300802702105

46 Malterud K. *Kvalitative forskningsmetoder for medisin og helsefag: en innføring*. Oslo: : Universitetsforlaget 2017.

47 Weekly epidemiological update on COVID-19 - 8 June 2021. https://www.who.int/publications/m/item/weekly-epidemiological-update-on-covid-19---8-june-2021 (accessed 12 Jun 2021).

48 Norwegian Institute of Public Health, (NIPH). Statistics coronavirus and covid-19 - Key figures for Norway. 2021.https://www.fhi.no/sv/smittsomme-sykdommer/corona/dags--og-ukerapporter/dags--og-ukerapporter-om-koronavirus/ (accessed 28 Apr 2021).

For peer review only

Table 1 Description of the participants of the study and characteristics of the interview situation.

Person	*Age range*	*DM Duration years*	*A1c mmol/mol*	*Diabetes complications*	*COVID-19*[1]	*Occupation exposed*	*Living alone*	*Interview form*	*Interview duration minutes*	*Site*[2]
1	60s	46	61	yes			no	meeting	56	1
2	40s	23	64	yes			no	video	25	1
3	30s	23	51	yes			yes	meeting	51	2
4	50s	45	66	yes		Kindergarten	yes	video	39	2
5	20s	17	60	no			no	video	17	2
6	40s	3	63	no	(+)		no	video	33	2
7	50s	19	54	no			no	video	59	1
8	20s	25	50	no			no	video	43	3
9	50s	21	51	no	+		no	video	38	3
10	50s	14	66	no	+		no	video	42	3
11	50s	9	63	no			no	video	22	3
12	40s	29	53	yes		School assistant	no	video	42	3
13	30s	20	65	no			yes	telephone	57	3
14	20s	10	46	no		Healthcare	yes	video	27	3
15	50s	53	57	yes			no	video	35	2
16	40s	44	78	no			no	telephone	45	2
17	40s	37	53	no		Teacher	no	video	36	2
18	50s	32	48	yes			no	telephone	93	2
19	50s	26	53	no			yes	video	51	3
Mean	**45 years**	**26 years**	**58 mmol/mol**						**43 min**	

[1] The symbol + denotes a confirmed case of COVID-19 infection, and the symbol (+) denotes suspected but unconfirmed infection.

[2] The three different sites of the study are represented by numbers 1, 2 and 3. The total patient populations at the three hospitals were 160 000 (1), 240 000 (2) and 560 000 (3). According to the annual report from the Norwegian Diabetes Register for Adults, 393 (1), 699 (2) and 1230 (3) patients with type 1 diabetes are treated and followed up at these outpatient clinics.

Table 2 Interview guide for the study, including probing questions and background information.

TOPIC	Q1	Q2	Q3	Q4	Q5	Q6	**Probing questions**	**Background information**
**Topic1: COVID-19 and daily life and coping with the situation**	When you first heard about the COVID-19 virus and that it had spread to Norway, what were your thoughts?	Do you have any thoughts about whether this virus is dangerous for you, and if so, how have these thoughts affected you?	Have you taken any specific actions to take care of your diabetes treatment during this period?	Can you describe how your everyday life is or was affected by the COVID-19 pandemic?	Can you describe what it was like for you and your family when society started reopening?	Can you describe what it has been like for you to take care of the diabetes treatment during this time?	Where did this happen?<br><br>What did you do in this situation / can you explain more about what happened?<br><br>What did you think in this situation?<br><br>What feelings did you have in this situation?<br><br>What do you think was the most demanding part of this situation?<br><br>What does this mean for you in the future?	For how long have you had diabetes?<br><br>Have you developed any diabetes complications?<br><br>What was your last registered A1c?
**Topic 2: Sources of information about COVID-19 and diabetes**	How did you and your family relate to news and media coverage of the pandemic?	Can you describe how you got information about the COVID-19 pandemic?						
**Topic 3: COVID-19 and help from the healthcare service**	What expectations have you had about possible help / support from the health service to cope with the situation?	Do you feel that your expectations have been met?	If there was a new pandemic, what kind of health service would you wish to be established for people in risk groups?					

For peer review only

Table 3 Data analysis of experiences from quotations, grouped by subcategory, category and main area.

QUOTATION	SUBCATEGORY	CATEGORY	MAIN AREA
"Because it was so uncertain whether I was more at risk or, uhm yes, or how things were because no one knew that much. So, I thought it was scary." (3)	Uncertainty caused by belonging to a high-risk group	Concerns related to COVID-19 and T1DM	Increased psychosocial burden of T1DM during the COVID-19 pandemic
"But I was genuinely afraid of getting this, because I thought that if I get it, I'll die." (9)	Anxiety concerning infection		
"The worst thing about the corona, when it came, it was all the uncertainty – we knew nothing." (18)	Concerns about the overall situation		
"I paid attention at first, and then I thought this is not good, I will get depressed from seeing this, all this intimidating propaganda." (19)	Continuous media focus raised concerns	A need for knowledge about T1DM and COVID-19	
"If I've been missing anything to some extent, it might getting insight into why diabetics are particularly vulnerable in a corona situation." (10)	Need for balanced information for people in risk groups		
"I contacted the doctor's office. I also contacted the hospital to find out if I was at risk and what to do if called back to work." (4)	A desire for personalized information		
"No, it's more the social discomfort you feel when people you meet sort of go for a hug and then you have to say 'You know what? I don't do that'." (14)	Challenges in social distancing	Consequences in social relations from COVID-19-related restrictions	
"And it was – especially for the children – it was probably the most demanding, that they had to avoid being with others for such a long time." (10)	Increased restrictions for family members of people with T1DM		
"So, it was kind of that I should not return to work, so that they [her colleagues] would not get a bad conscience if I were to die." (4)	Restrictions related to being in a high-risk group due to T1DM		
"I felt, when we locked down, that … that the start was completely awful because it, uhm, it completely changed my routines." (3)	Difficulties creating routines in everyday life at home	Altered routines due to COVID-19 affected T1DM self-management	Changed conditions for T1DM treatment during the COVID-19 pandemic

For peer review only - http://bmjopen.bmj.com/site/about/guidelines.xhtml

"Uhm yes, but it makes one stay indoors more and such, and then it's easier to make a little extra-good food so, …" (2)	Difficulties maintaining a healthy lifestyle	
"Surprisingly, the treatment has been better than ever before. But it has to do with the fact that I got a new type of insulin pump. And it has really made everyday life a lot easier." (8)	The benefit of glucose sensor and insulin pump in diabetes treatment	
"I had an incredibly high fever and had an insanely high blood glucose." (9)	Challenging fluctuations in blood glucose during COVID-19 disease	
"It [the blood glucose] has been affected because I, well it's much busier at work [teacher] because we wash desks and surfaces all the time." (17)	New routines at work to prevent the spread of COVID-19	Changes in the work or study situation due to COVID-19 affecting T1DM self-management
"But clearly, the lack of breaks and not being able to eat and such, I must say that I have sometimes felt that it has been tough at work." (17)	Increased workload due to COVID-19 caused stress	
"All the time I feel that I have kind of a 'sting' in the back of my mind, whether I might be laid off again." (18)	Financial uncertainty caused stress	
"It's even easier, when I work from home, to maintain good blood glucose control, compared with running around taking those measurements at work all the time." (10)	More predictable everyday life gave more time to focus on T1DM self-management	
"The one [health check] before the summer was just completely eliminated, so there was a year in between check-ups and I don't think that is good." (13)	Lack of or delayed follow-up from the specialist health service	Reorganization of the health service due to COVID-19 affecting T1DM treatment
"I must admit that in the beginning I thought 'This is a state of emergency, now I have to stock up on insulin'." (15)	Concerns regarding access to medicines	
"And if I should end up with someone who does not necessarily have full control over what blood glucose is all about in such an intensive ward, then yes. (7)	Concerns about ICU healthcare professionals' knowledge of specialised diabetes treatment in case of severe illness and need for help	
"In the end I called the diabetes nurse and asked: 'What am I going to do? I can't get my blood glucose down'." (9)	Availability of healthcare professionals by telephone	

For peer review only

Table 4 Data analysis of actions from quotations, to subcategories, via categories up to the main areas.

QUOTATION	SUBCATEGORY	CATEGORY	MAIN AREA
"I was very, very conscious about keeping my distance to others, and as I said, I wore a mask as well." (18)	Increased awareness of complying with infection control advice	Practical strategies to avoid being infected with COVID-19	Actions to handle psychosocial strain related to T1DM and COVID-19
"Every Saturday he [her brother] goes to the store and buys groceries for me." (15)	Getting help from others to obtain food and medicine		
"We can't let you [the children] take the bus unnecessarily, you should not go to a party where there are 50 people, that is not what we should do now. So yes, they must consider me." (16)	Limiting family members' number of social contacts		
"I have a good friend who has bone marrow cancer. And we both agreed to it because she is very restrictive about who she socializes with too, so the two of us had a lot of contact." (4)	Choosing a few people to meet and socialize with	Measures to maintain social contact with others	
"And in this hobby community they are good at organizing such online meetings, so we sit at our PCs together instead of alone." (12)	Creating alternative arenas for socialization		
"I'm so fortunate that I have a husband that I live with. I'm married, you know, so I think that the loneliness that many feel. I'm much better off." (15)	Finding joy and support within the family		
"And then I thought well, it's not certain that it's really bad that I get it [COVID-19] because I'm pretty healthy, I have a good Hba1c, my immune system is kind of good." (5)	Positive cognitive reflections to deal with the situation	Mental strategies to deal with the situation	
"I kind of tried to avoid maybe the newspapers as much as possible, because I feel that … well, it affects me in a negative sense." (3)	Selective attitude towards media information		
"I asked my doctor what should I do? Should I just continue working from home and answer technical questions, or should I travel and work as normal?" (19)	Actively searching for personalized information		

For peer review only - http://bmjopen.bmj.com/site/about/guidelines.xhtml

"I quickly decided that I must live as normally as possible. Eat as normal. Be physically active outside. Keep in touch with others in a safe way." (4)	Creating predictable everyday routines for T1DM self-management	Creating a safe structure in daily life	Actions to handle changed conditions for T1DM treatment during the COVID-19 pandemic
"So then I eat in the car- instead of going in there to eat with the others [on a construction site]." (2)	Starting new routines at work for infection control		
"I've been pretending that it's a normal day. I get up at regular times, shower, put on make-up, etc." (15)	Creating appropriate home-office routines		
"And I have enough things to do so that there will not be much effort spent on thinking too much, to put it that way." (11)	Consciously finding meaningful pursuits to avoid brooding		
"And also I think we are very fortunate to have the diabetes system we have here in Norway; that I was allowed to get Freestyle Libre and such." (10)	Using medical technical equipment actively in treating diabetes	Measures to ensure proper T1DM self-management during the COVID-19 pandemic	
"I could not go to the gym, so I had to use nature instead." (4)	Alternative physical activities to maintain a healthy lifestyle during the COVID-19 pandemic		
"And tried to inject, took higher and higher insulin doses." (9)	Titrate insulin doses in relation to blood glucose fluctuations during COVID-19 disease		
"Then I was isolated in the bedroom by my wife. I was not allowed to go out." (9)	Isolating due to COVID-19 illness		
"And about medicine and stuff. I have always had a stock of insulin." (4)	Keeping a stockpile of insulin available for emergencies		

COREQ (COnsolidated criteria for REporting Qualitative research) Checklist

A checklist of items that should be included in reports of qualitative research. You must report the page number in your manuscript where you consider each of the items listed in this checklist. If you have not included this information, either revise your manuscript accordingly before submitting or note N/A.

Topic	Item No.	Guide Questions/Description	Reported on Page No.
**Domain 1: Research team and reflexivity**			
Personal characteristics			
Interviewer/facilitator	1	Which author/s conducted the interview or focus group?	
Credentials	2	What were the researcher's credentials? E.g. PhD, MD	
Occupation	3	What was their occupation at the time of the study?	
Gender	4	Was the researcher male or female?	
Experience and training	5	What experience or training did the researcher have?	
Relationship with participants			
Relationship established	6	Was a relationship established prior to study commencement?	
Participant knowledge of the interviewer	7	What did the participants know about the researcher? e.g. personal goals, reasons for doing the research	
Interviewer characteristics	8	What characteristics were reported about the inter viewer/facilitator? e.g. Bias, assumptions, reasons and interests in the research topic	
**Domain 2: Study design**			
Theoretical framework			
Methodological orientation and Theory	9	What methodological orientation was stated to underpin the study? e.g. grounded theory, discourse analysis, ethnography, phenomenology, content analysis	
Participant selection			
Sampling	10	How were participants selected? e.g. purposive, convenience, consecutive, snowball	
Method of approach	11	How were participants approached? e.g. face-to-face, telephone, mail, email	
Sample size	12	How many participants were in the study?	
Non-participation	13	How many people refused to participate or dropped out? Reasons?	
Setting			
Setting of data collection	14	Where was the data collected? e.g. home, clinic, workplace	
Presence of non-participants	15	Was anyone else present besides the participants and researchers?	
Description of sample	16	What are the important characteristics of the sample? e.g. demographic data, date	
Data collection			
Interview guide	17	Were questions, prompts, guides provided by the authors? Was it pilot tested?	
Repeat interviews	18	Were repeat inter views carried out? If yes, how many?	
Audio/visual recording	19	Did the research use audio or visual recording to collect the data?	
Field notes	20	Were field notes made during and/or after the inter view or focus group?	
Duration	21	What was the duration of the inter views or focus group?	
Data saturation	22	Was data saturation discussed?	
Transcripts returned	23	Were transcripts returned to participants for comment and/or	

For peer review only

Topic	Item No.	Guide Questions/Description	Reported on Page No.
		correction?	
**Domain 3: analysis and findings**			
Data analysis			
Number of data coders	24	How many data coders coded the data?	
Description of the coding tree	25	Did authors provide a description of the coding tree?	
Derivation of themes	26	Were themes identified in advance or derived from the data?	
Software	27	What software, if applicable, was used to manage the data?	
Participant checking	28	Did participants provide feedback on the findings?	
Reporting			
Quotations presented	29	Were participant quotations presented to illustrate the themes/findings? Was each quotation identified? e.g. participant number	
Data and findings consistent	30	Was there consistency between the data presented and the findings?	
Clarity of major themes	31	Were major themes clearly presented in the findings?	
Clarity of minor themes	32	Is there a description of diverse cases or discussion of minor themes?	

Developed from: Tong A, Sainsbury P, Craig J. Consolidated criteria for reporting qualitative research (COREQ): a 32-item checklist for interviews and focus groups. *International Journal for Quality in Health Care*. 2007. Volume 19, Number 6: pp. 349 – 357

Once you have completed this checklist, please save a copy and upload it as part of your submission. DO NOT include this checklist as part of the main manuscript document. It must be uploaded as a separate file.

For peer review only

BMJ Open

Experiences and actions related to living with type 1 diabetes during the COVID-19 pandemic in Norway - A qualitative study conducted during July - December 2020

Journal:	*BMJ Open*
Manuscript ID	bmjopen-2021-056027.R1
Article Type:	Original research
Date Submitted by the Author:	07-Dec-2021
Complete List of Authors:	Pleym, Karin; Western Norway University of Applied Sciences, Department of Health and Caring Sciences; Vestre Viken Hospital Trust, Department of Medicine<br>Iversen, Marjolein; Western Norway University of Applied Sciences, Department of Health and Caring Sciences<br>Broström, Anders; Jönköping University, Department of Nursing, School of Health and Welfare; Linköping University Hospital, Department of Clinical Neurophysiology
**Primary Subject Heading**:	Diabetes and endocrinology
Secondary Subject Heading:	Nursing, Qualitative research
Keywords:	COVID-19, QUALITATIVE RESEARCH, DIABETES & ENDOCRINOLOGY

SCHOLARONE™
Manuscripts

I, the Submitting Author has the right to grant and does grant on behalf of all authors of the Work (as defined in the below author licence), an exclusive licence and/or a non-exclusive licence for contributions from authors who are: i) UK Crown employees; ii) where BMJ has agreed a CC-BY licence shall apply, and/or iii) in accordance with the terms applicable for US Federal Government officers or employees acting as part of their official duties; on a worldwide, perpetual, irrevocable, royalty-free basis to BMJ Publishing Group Ltd ("BMJ") its licensees and where the relevant Journal is co-owned by BMJ to the co-owners of the Journal, to publish the Work in this journal and any other BMJ products and to exploit all rights, as set out in our licence.

The Submitting Author accepts and understands that any supply made under these terms is made by BMJ to the Submitting Author unless you are acting as an employee on behalf of your employer or a postgraduate student of an affiliated institution which is paying any applicable article publishing charge ("APC") for Open Access articles. Where the Submitting Author wishes to make the Work available on an Open Access basis (and intends to pay the relevant APC), the terms of reuse of such Open Access shall be governed by a Creative Commons licence – details of these licences and which Creative Commons licence will apply to this Work are set out in our licence referred to above.

Other than as permitted in any relevant BMJ Author's Self Archiving Policies, I confirm this Work has not been accepted for publication elsewhere, is not being considered for publication elsewhere and does not duplicate material already published. I confirm all authors consent to publication of this Work and authorise the granting of this licence.

For peer review only

Experiences and actions related to living with type 1 diabetes during the COVID-19 pandemic in Norway - A qualitative study conducted during July - December 2020

Karin Pleym RN, MSc[1,2], Marjolein M. Iversen PhD[1] and Anders Broström Phd[3,4]

1. Faculty of Health and Social Science, Western University of Applied Sciences, Norway

2. Department of Medicine, Vestre Viken Drammen Hospital, Norway

3. Department of Nursing, School of Health and Welfare, Jönköping University, Jönköping, Sweden

4. Department of Clinical Neurophysiology, Linköping University Hospital, Linköping, Sweden

Corresponding author: Marjolein M. Iversen, Inndalsveien 28, 5063 Bergen, Norway. Tel. +47 902 55 044, e-mail: Marjolein.Memelink.Iversen@hvl.no

Word count:

Abstract: 298 words

Manuscript: 4160

Tables: 4

Figures: 0

References: 50

For peer review only

Abstract

Objectives: The aim of this study was to describe the experiences of adults with type 1 diabetes (T1DM) during the COVID-19 pandemic in Norway, and what actions they took to cope with the situation.

Design: An inductive, descriptive design applying the critical incident technique (CIT) was used to collect qualitative data between July 2020 and December 2020.

Setting: A strategic selection was made from diabetes specialist outpatient clinics at three different hospitals in eastern Norway. The hospitals, two community hospitals and one university hospital, were situated in both rural and urban areas.

Participants: Inclusion criteria were people with T1DM aged 18–65 years. Exclusion criteria were pregnancy, people with chronic pulmonary disorders, people with active cancer diseases and people diagnosed with a myocardial infarction or stroke during the previous six months. Semi-structured individual interviews with 19 people with T1DM were conducted.

Results: Experiences were categorized into two main areas: "Increased psychosocial burden of T1DM during the COVID-19 pandemic" and "Changed conditions for T1DM treatment during the COVID-19 pandemic". Uncertainty distress and social consequences from infection control measures contributed to the burden of T1DM. Disrupted T1DM follow-up and altered daily routines created challenges. However, having increased time to focus on T1DM self-management during lockdown represented an improvement. Actions to handle the situation were categorized into two main areas: "Actions to handle psychosocial strain related to T1DM and COVID-19" and "Actions to handle changed conditions for T1DM treatment during the COVID-19 pandemic".

Conclusions: Patients experienced an increased psychosocial burden of T1DM and difficulties from a disrupted daily life affecting T1DM self-management routines. Uncertainty-reducing behaviours and actions to adapt to the situation provided a general sense of coping despite these difficulties. Tailored information and follow-up by telephone or video-call was emphasized to reduce uncertainly distress and support adequate diabetes T1DM self-management.

For peer review only

Strengths and limitations of this study

- Three researchers with good knowledge of both diabetes and critical incident technique have been involved throughout the study.
- Utilising the critical incident technique, we were able to focus on both experiences and actions used by people with type 1 diabetes during the COVID-19 pandemic in Norway.
- The majority of the interviews were performed by video-call giving the participants the comfort of familiar environments and contributing to a safe interview situation.
- A possible paradox in the study may be that subjects who suffer from lock-down, usually could be the ones who want to participate in the study.
- Norway had an early complete lock-down prior to the data collection, and relatively few deaths from COVID-19 compared to other European countries.

For peer review only

INTRODUCTION

The outbreak of SARS-CoV-2-induced coronavirus disease 2019 (COVID-19) was declared a global pandemic by the World Health Organization on 11 March 2020.[1] The COVID-19 disease ranges in severity from asymptomatic or mild cases to severe illness and death.[2,3] Diabetes has been identified as one of several underlying health conditions associated with increased severity and higher mortality rates from COVID-19 illness.[4–9]

In Norway the government implemented strict interventions to limit the spread of the virus.[10] The infection control measures had significant impacts on the population's everyday life. Previous reports from preceding coronavirus epidemics such as SARS-CoV (SARS) in 2002–2003 and the Middle East Respiratory Syndrome coronavirus (MERS) in 2012–2015 showed evidence of increased psychosocial and mental health challenges.[11–13]

Similarly, psychosocial challenges caused by the COVID-19 pandemic have been seen in the general population.[14–16] The massive media coverage and the general perception of the severity of the situation had potential to cause stress and anxiety. People with type 1 diabetes (T1DM) can be more vulnerable to stress in terms of a direct effect of stress hormones on blood glucose levels and an indirect effect on the person's behaviour related to diabetes self-management.[17] Previous studies have shown that people with T1DM experienced disrupted follow-up from specialist outpatient clinics during the COVID-19 pandemic and challenges affecting T1DM self-management routines.[18–22] Other studies, however, showed improved glycaemic control in periods of lockdown of society.[23–25] A cross-sectional survey from Denmark, in which 2 430 adults with diabetes completed an online questionnaire, indicated that people with diabetes have COVID-19-specific worries about their diabetes associated with poorer psychosocial health.[26] In line with these results, diabetes nurses across Europe have reported a significant increase in physical and psychological problems in patients with diabetes.[27] A qualitative study has provided insight into the experiences of people diagnosed with various types of diabetes in the initial phase of lockdown in Denmark.[28] However, little is known about how people with T1DM handled daily life during the pandemic over a longer period of time that includes the reopening of society. Knowledge of the experiences and actions of people with T1DM during

For peer review only

the COVID-19 pandemic may provide insight into strategies for handling the situation as well as possible needs for assistance from the healthcare system. This knowledge may in turn be valuable for other risk groups and in the event of similar situations in future. The aim of this study was to describe the experiences of adults with T1DM during the COVID-19 pandemic in Norway and what actions they took to cope with their situation.

RESEARCH DESIGN AND METHODS

Design and method description

This qualitative study used an inductive, descriptive design, applying the critical incident technique (CIT).[29] A "critical incident" represents a major event of great importance to the person involved, and is an essential part of the CIT methodology. Critical incidents consist of positive or negative experiences in addition to actions carried out to handle the phenomenon in question.[30] Due to its flexibility and the focus on the patient perspective, CIT is considered an eligible method for use in healthcare research.[31] The method enables informants to describe significant incidents as well as their experiences and actions. Consequently, CIT is a suitable method for exploring the research question of this study.

Ethical considerations

The Regional Committee for Medical and Health Research Ethics in South East Norway (REC-154546), the Norwegian Centre for Research Data (NSD) (NSD-39284), the local data protection officers at the three study sites and Western Norway University of Applied Science evaluated the study protocol and accepted it before study start.

Participants and setting

The informants were recruited from the diabetes specialist outpatient clinics at three different hospitals in eastern Norway. The hospitals, two community hospitals and one university hospital, were situated in both rural and urban areas. The total patient

For peer review only

populations at the hospitals were 160 000, 240 000 and 560 000 respectively. People with T1DM will typically meet with a diabetes nurse or an endocrinologist in the diabetes outpatient clinics every three to six months.

A strategic selection was made to ensure a heterogeneous group of informants. Inclusion criteria were people with T1DM aged 18–65 years. Exclusion criteria were pregnancy, people with chronic pulmonary disorders, people with active cancer diseases and people diagnosed with a myocardial infarction or stroke during the previous six months. Characteristics such as gender, age, T1DM duration, metabolic control and having an occupation with increased exposure to possible infection were used in the selection of informants (Table 1). Based on the criteria for strategic selection, a local designated diabetes nurse provided oral information about the study to potential participants. When interest was expressed, the nurse asked for oral consent to be contacted by the first author by telephone to provide further information about the study. The first author then made contact by telephone and gave additional information, and written information was sent to the potential participant by mail. After written informed consent was returned to the first author, an appointment for an interview was made. Three informants later cancelled the interview appointment due to a busy schedule (2) or illness (1). In total, 19 people with T1DM were included in the study.

-Insert Table 1

Data collection

Descriptions of experiences and actions were obtained through semi-structured individual interviews. Due to the ongoing pandemic, informants could choose to conduct the interviews in person, by video call or by telephone. The video interviews were conducted on an encrypted web platform.

A semi-structured interview was performed based on an interview guide. The interview guide was developed by all authors together, who have extensive clinical and methodological competence. A user representative from the local diabetes association reviewed the interview guide and gave advice on the content. After three interviews were conducted, the transcriptions were discussed within the research team. The interview guide consisted of three main topics and probing questions as presented in Table 2.

For peer review only

The interviews were conducted between July 2020 and December 2020. Norway was in complete lockdown from March 12th to mid-April 2020. After this, a cautious reopening of society was initiated. Nevertheless, the authorities' advice on social distancing and hygiene measures was maintained. In July 2020, when the first interviews were conducted, the community was relatively open. Later in the autumn, infection rates started to increase again, which led to a stronger focus on measures such as working from home, partially closed schools, the obligation to wear masks and so forth. There was no current Sars-Cov2 vaccine available in Norway at the time of this study.

The interviews were all performed by the first author and recorded on a digital recorder. The first author transcribed 12 and a project assistant transcribed seven of the audio recordings verbatim.

-Insert Table 2

Data analysis

The study was analysed in line with CIT as described by Fridlund et al.[30] Data were regularly discussed in the research team until consensus regarding categorization was achieved among the three authors. The first author read the interviews several times to obtain an overall picture of the content. Experiences and actions related to the purpose of the study were highlighted in the text and delimited. A total of 313 experiences and 185 actions was identified and categorized. Delimitations from the text were then compared for similarities in an effort to group them into subcategories. When the subcategories were identified, a new process of exploration and comparison was performed to group the subcategories into categories. The purpose of a category is to describe the general character of the subcategories. Following the establishment of categories, a new effort was made to group categories into main areas. The purpose of constructing main areas was to describe the overall theme of the data. Descriptions of experiences and actions were analysed separately. This process led to the finding of 21 subcategories of experiences and 18 subcategories of actions. The subcategories were then structured into six categories of experiences and five categories of actions. Finally, two main areas of experiences and actions were identified.

For peer review only

RESULTS

In total, 10 female and nine male people with T1DM participated in the study, of which two had been diagnosed with COVID-19. All the informants were Norwegian born with the same cultural background (Table 1).

Experiences

Table 3 presents the experiences of people with T1DM during the COVID-19 pandemic in Norway, including direct quotations from the interviews. Two main areas emerged from the data analysis of experiences: "Increased psychosocial burden of T1DM during the COVID-19 pandemic" and "Changed conditions for T1DM treatment during the COVID-19 pandemic".

-Insert Table 3

Increased psychosocial burden of T1DM during the COVID-19 pandemic

Concerns related to COVID-19 and T1DM

Informants described uncertainty caused by belonging to a risk group. This included fear of getting sick from COVID-19 and confusion about the reasons why T1DM was associated with higher risk of a fatal outcome. General anxiety about the uncertainty of the overall situation was described as difficult.

A need for knowledge about T1DM and COVID-19

The continuous media coverage caused anxiety and worries. The informants expressed a need for balanced information aimed at people in risk groups. Additionally, personalized information was desired and sought by contacting familiar healthcare providers such as the general practitioner or the diabetes nurse.

For peer review only

Consequences for social relations as a result of COVID-19-related restrictions

This category describes the various difficulties the informants experienced while socializing with family, friends, colleagues and others. To avoid bringing the COVID-19 virus into the home, family members of people with T1DM were subjected to stricter restrictions than their peers. For instance, the children of one informant were not allowed to use public transport, and the son of a divorced woman moved temporarily to live with his father. Experiences of social limitations in the workplace due to the T1DM diagnosis were reported. For example, one of the informants was told that it was not desirable for her to return to the workplace after the termination of lockdown, as colleagues were afraid of infecting her.

Changed conditions for T1DM treatment during the COVID-19 pandemic

Altered routines due to COVID-19 affecting T1DM self-management

Restrictions implemented by the government to prevent the spread of COVID-19 created changes in daily routines which in turn affected T1DM self-management. Informants expressed difficulties in maintaining a healthy lifestyle in terms of physical activity and healthy diet. To have a good time within the family, food such as cakes and ice cream were served more frequently. Since the gyms were closed, less familiar activities were initiated which made it more difficult to predict correct insulin doses. Informants who contracted COVID-19 experienced large fluctuations in and high levels of blood glucose during the period of illness. Having access to continuous blood glucose monitoring (CGM) and insulin pumps was seen as an advantage in handling this situation. Reaching out to the diabetes nurse by telephone for advice on how to titrate insulin doses gave a sense of security.

Changes in the work or study situation due to COVID-19 affecting T1DM self-management

Measures to prevent infection in the workplace led to new routines. This had an impact on meal breaks, time spent being physically active and opportunities for social contact at work. Some, such as a teacher and a mail delivery worker, experienced increased work pressure and new work tasks, whereas others experienced a more predictable daily life with less stress and more time to take care of T1DM self-management. Being laid off from work

For peer review only - http://bmjopen.bmj.com/site/about/guidelines.xhtml

because of lockdown gave rise to financial insecurity. This led to stress which in turn affected blood glucose levels.

Reorganization of the health service due to COVID-19 affecting T1DM-treatment

Informants experienced delayed follow-up from the specialist outpatient clinic. This was due to the reorganization of hospital resources to prepare for a large number of COVID-19 patients. The authorities introduced a temporary rationing of insulin to prevent hoarding and ensure access. This led to concerns about the availability of medications and created worries about the inconvenience of having to go to the pharmacy more frequently. Informants' concerns about possible lack of competence in the intensive care unit regarding specialized T1DM treatment made the risk of becoming severely ill from COVID-19 even more frightening. These concerns related to previous experiences from hospitalizations. Having experienced easy access to healthcare professionals before the pandemic made them feel confident that they could reconnect for information and advice when needed.

Actions

Table 4 presents the actions of people with T1DM during the COVID-19 pandemic in Norway, including direct quotations from the interviews. The two main areas of actions that emerged from the data analysis were "Actions to handle psychosocial strain related to T1DM and COVID-19" and "Actions to handle changed conditions for T1DM treatment during the COVID-19 pandemic". The analysis of actions is described in Table 4.

-Insert Table 4

Actions to handle psychosocial strain related to T1DM and COVID-19

Practical strategies to avoid being infected with COVID-19

Informants described increased awareness of complying with general advice on infection control. Measures such as social distancing and frequent hand-washing became a daily routine. Some started using a mask early on. Avoiding crowded places and receiving help

For peer review only

from others for grocery shopping and providing necessary medications were strategies adopted to prevent infection. Limiting the number of social contacts for the family as a whole was considered necessary to avoid infection.

Measures to maintain social contact

Informants described finding support and joy within the family. Choosing a few people to meet regularly gave them the opportunity to maintain social contact outside the home. Children of parents with T1DM were allowed to choose one or two friends to play with. Both new and familiar digital platforms served as alternative arenas for social interaction.

Mental strategies to deal with the situation

Informants sought personalized information about their risk status. Positive thinking was one of the main strategies to cope with the overall situation. Furthermore, informants actively avoided media coverage of COVID-19.

Actions to handle changed conditions for T1DM treatment during the COVID-19 pandemic

Creating a safe structure in daily life

Predictable routines were created to facilitate T1DM self-management. New job routines were implemented to protect the informants from being infected. Working from home was challenging, but establishing home-office routines was experienced as a positive factor. Lack of routine made handling work and T1DM self-management difficult. Meaningful activities at home were found helpful to avoid pondering about the situation.

Measures to ensure proper T1DM self- management during the COVID-19 pandemic

Medical devices such as CGM and insulin pumps were perceived as useful in the treatment of T1DM and contributed to good metabolic control. Furthermore, informants found alternative physical activities to maintain a healthy lifestyle when the fitness centres were

For peer review only

closed. Keeping a storage of insulin led to a sense of security. For those who contracted COVID-19, high blood glucose levels caused by the infection were met with efforts to titrate insulin doses, and informants called the diabetes nurse for support and advice on this matter.

CONCLUSION

This study aimed to describe the experiences and actions of people with T1DM during the COVID-19 pandemic in Norway between July and December 2020, when no current Sars-Cov2 vaccine was available in Norway. Results indicate that the psychosocial burden of living with T1DM increased during the pandemic. Uncertainty caused by belonging to a risk group, fear of severe illness and strict adherence to social distancing for the family as a whole contributed to the burden of T1DM. Additionally, we found that infection control measures and reorganization of the healthcare system represented challenges in the form of changed conditions for T1DM treatment. Applying manageable, understandable and meaningful actions to deal with the psychosocial strain and the disrupted daily life led to a general sense of coping despite difficulties.

Alteration of daily routines brought challenges such as an increased amount of unhealthy food and less exercise. These findings are in line with previous quantitative research.[32,33] In a Saudi cross-sectional study of people with T1DM using an insulin pump, difficulties in maintaining a healthy diet and staying physically active during lockdown were seen. However, adherence to the insulin pump therapy contributed to stabilizing blood glucose.[32] Participants in our study described that general stress and anxiety affected T1DM self-management in terms of fluctuations in blood glucose similar to those seen in studies from Italy.[22,34] Nevertheless, our participants also described a more predictable lifestyle in working from home that led to improved control of blood glucose levels. Similar findings of improved metabolic control have been reported from studies of children with T1DM on insulin pump therapy.[35–38] Furthermore, multiple studies that reviewed metabolic control via CGM data registered during the pandemic found improvements in

For peer review only

both time in range and blood glucose levels,[23–25] although one study showed a tendency of increased fluctuations in physical activity and perceived stress.[34]

The finding of an increased psychosocial burden of T1DM during the COVID-19 pandemic is consistent with existing evidence from quantitative studies.[26,27] Our study provides an in-depth understanding of these psychosocial difficulties, and is consistent with a qualitative study conducted in Denmark.[28] While the Danish study focused on the experiences of people with multiple types of diabetes during lockdown in Denmark (April 2020), the results from our study focused on people with T1DM at a later stage of the pandemic (July 2020–December 2020). Therefore, we covered experiences related to the reopening of society as well as to new periods of lockdown. In comparison with the Danish study, our findings may indicate that the psychosocial burden of T1DM during the pandemic increased over time. This interpretation is supported by another qualitative study of people living with different long-term physical health conditions during the COVID-19 pandemic. In this study they found that the COVID-19 pandemic affected several aspects of mental health and well-being.[16] Furthermore, in a review of the effects of quarantine, impacts on psychosocial health were seen, and the length of the period of isolation affected the outcomes of these negative effects.[12] Even though our participants were not quarantined, they were to some extent self-isolating for extended periods, and thus the psychosocial burden might have increased accordingly.

"Diabetes distress" refers to a psychological condition associated with the burden of living with T1DM.[39] Diabetes distress might add to general stress from the severity of the pandemic situation and further increase the psychosocial burden of T1DM. In a previous study, diabetes distress was identified as one of several factors associated with poorer psychosocial health in patients with diabetes during the COVID-19 pandemic.[26]

Freeston et al.[40] have proposed a model of uncertainty distress in the context of the COVID-19 pandemic. According to this model, uncertainty can be defined as “the subjective negative emotions experienced in response to the as yet unknown aspects of a given situation.”[40] The degree of uncertainty distress depends on the following factors: disruption of everyday life, perceived actual threat and perceived uncertainty. In addition, individual tolerance of uncertainty influences uncertainty distress. Individual tolerance of uncertainty can be modified by uncertainty-reducing behaviours. Such behaviours may

For peer review only

include actions such as avoidance, preparation for possible negative consequences, postponement and the search for reassurance. The actions taken by the participants in our study can be seen in the light of this model of uncertainty distress in the context of the COVID-19 pandemic. Our participants actively avoided media, they prepared for possible negative consequences by keeping a stock of insulin and by strictly complying with advices on infection control measures. Furthermore, social gatherings were postponed, and the participants searched for tailored and personalized information for reassurance.

In this study, we have described how various actions were taken by the participants to adapt, and positive thinking was one of the main strategies to cope with the overall situation. From the perspective of the theory of salutogenesis, stressors in life will not necessarily contribute to poor outcomes of health if met properly. The essential factor is that the individual finds understandable, manageable and meaningful actions to adapt. Through meaningful actions, people may achieve a sense of coherence and thus of coping despite challenging environments.[41–43]

Participants in our study emphasized the importance of access to healthcare professionals by telephone for personalized guidance. A recently published but not yet peer-reviewed study from Norway shows a surprising and significant increase in non-COVID-related deaths in patients with diabetes from March 2020 to May 2020.[44] The causal factors behind these findings are unknown, and it is not possible to draw conclusions. Nevertheless, Raknes et al. propose inappropriate avoidance of healthcare professionals and thus a lack of follow-up as a possible explanation. Based on this understanding, the importance of having healthcare professionals such as diabetes nurses available by telephone for medical advice and social support is further underlined.

Study limitations

We have strived to achieve trustworthiness throughout the study in terms of credibility, dependability, confirmability and transferability as described by Polit & Beck.[45]
Three researchers with good knowledge of both CIT and diabetes have been involved throughout the study. The first is a diabetes specialist nurse, she has been involved in all

For peer review only

stages of this research project. The two others are professors in nursing with extensive methodological knowledge of CIT.

The first author has clinical experience which includes answering calls from diabetes patients anxious about COVID-19. Her experience represents a factor that might threaten reflexivity. On the other hand, substantial knowledge from the field can be a valuable source of relevant and specific research.[46] The first author's clinical experience can be seen as an advantage in the process of formulating relevant questions for the interview guide. Awareness of the potential influence of previous experiences has been taken into account in the interpretation of the data. Description of the data has sought to be clear and has been discussed in depth within the research team to strengthen the credibility of the study. The interviews were all performed by the first author, which increased the likelihood that the interview guide was used in the same way in all the interviews. Out of 19 interviews, 14 were conducted by video call and three were conducted by telephone, which may have contributed to a loss of some nonverbal data.[47] On the other hand, participants had the comfort of familiar environments and did not have to worry about possible infection. We have provided a detailed description of the participants as important contextual factors regarding the dependability of the study (Table 1). In sampling, we aimed to achieve variation in gender, age, metabolic control, T1DM duration and occupation. However, we were unable to include participants with a different cultural background, which is a limitation. A possible paradox in the study may be that subjects who suffer most from lock-down, could be the ones who want to talk and seek comfort from healthcare providers. To strengthen dependability and facilitate confirmability, the systematic procedure we followed in the data analysis is clearly presented with quotations categorised and finally grouped into main areas (Table 3 and Table 4). A diversity of experiences and actions were obtained from the interviews, and this is seen as a strength when using the CIT approach.[29]

According to Malterud, the field and the context in which the data material is acquired will always determine the scope of the knowledge in time and space.[48] From an international perspective, Norway has had relatively few deaths from COVID-19 compared to other European countries.[49] As of the 28 April 2021 there are 753 registered deaths out of 111 685 confirmed COVID-19 cases in a population of 5 391 369 inhabitants.[50] Participants

For peer review only

included in our study live in the eastern part of Norway close to the capital. The capital and surrounding areas are the part of Norway most affected by the COVID-19 pandemic in terms of lock-down restrictions. From this perspective, it would be fair to say that the participants in this study may have had experiences similar to people in countries with higher infection rates and deaths from COVID-19. A detailed description of the study context, the participants, method of data collection and the data analysis, including direct quotations from the interviews, are presented (Table 1, Table 2, Table 3 and Table 4) to give the reader the opportunity to determine the transferability of the findings.

Conclusion and implications for clinical practice

In conclusion, participants experienced psychosocial strain and an increased burden of T1DM. Furthermore, they described uncertainty-reducing behaviours and actions which provided a general sense of coping with handling a disrupted daily life during the COVID-19 pandemic despite difficulties.

Healthcare professionals should be aware that many people with T1DM might have lived under increased stress during the COVID-19 pandemic. Availability of healthcare professionals for personalized advice by telephone was emphasized as important to alleviate uncertainty distress, reduce diabetes distress and support adequate T1DM self-management. Access to telephone and video consultations should be implemented to ensure follow-up of people with T1DM in similar situations in future.

Further research is needed to investigate potential psychosocial impacts of the COVID-19 pandemic on people with T1DM from a family perspective. Exploring possible associations between diabetes distress, uncertainty distress and psychosocial burden was beyond the scope of this study. However, this would be an interesting topic for future research.

Competing interests: Karin Pleym has received for a contribution of NOK 10 000 from the regional Diabetes Association for the project.

For peer review only

Contribution: KP, AB and MMI designed the study. MMI facilitated data collection. KP collected the data. KP, AB and MMI contributed to data analysis KP, AB and MMI contributed to drafting the manuscript and read and approved the final manuscript.

Data availability statement: All data relevant to the study are included in the article or uploaded as supplementary information**.**

References

1 WHO. WHO Director-General's opening remarks at the media briefing on COVID-19 - 11 March 2020. 2020.https://www.who.int/director-general/speeches/detail/who-director-general-s-opening-remarks-at-the-media-briefing-on-covid-19---11-march-2020 (accessed 10 Mar 2021).

2 Chen G, Wu D, Guo W, *et al.* Clinical and immunological features of severe and moderate coronavirus disease 2019. *J Clin Invest* 2020;**130**:2620–9. doi:10.1172/JCI137244

3 Zhou F, Yu T, Du R, *et al.* Clinical course and risk factors for mortality of adult inpatients with COVID-19 in Wuhan, China: a retrospective cohort study. *The Lancet* 2020;**395**:1054–62. doi:10.1016/S0140-6736(20)30566-3

4 Zhu L, She Z-G, Cheng X, *et al.* Association of Blood Glucose Control and Outcomes in Patients with COVID-19 and Pre-existing Type 2 Diabetes. *Cell Metab* 2020;**31**:1068-1077.e3. doi:10.1016/j.cmet.2020.04.021

5 McGurnaghan SJ, Weir A, Bishop J, *et al.* Risks of and risk factors for COVID-19 disease in people with diabetes: a cohort study of the total population of Scotland. *Lancet Diabetes Endocrinol* 2021;**9**:82–93. doi:10.1016/S2213-8587(20)30405-8

6 Barron E, Bakhai C, Kar P, *et al.* Associations of type 1 and type 2 diabetes with COVID-19-related mortality in England: a whole-population study. *Lancet Diabetes Endocrinol* 2020;**8**:813–22. doi:10.1016/S2213-8587(20)30272-2

7 Xu L, Mao Y, Chen G. Risk factors for 2019 novel coronavirus disease (COVID-19) patients progressing to critical illness: a systematic review and meta-analysis. *Aging* 2020;**12**:12410–21. doi:10.18632/aging.103383

8 Huang C, Wang Y, Li X, *et al.* Clinical features of patients infected with 2019 novel coronavirus in Wuhan, China. *The Lancet* 2020;**395**:497–506. doi:10.1016/S0140-6736(20)30183-5

9 Kumar A, Arora A, Sharma P, *et al.* Is diabetes mellitus associated with mortality and severity of COVID-19? A meta-analysis. *Diabetes Metab Syndr Clin Res Rev* 2020;**14**:535–45. doi:10.1016/j.dsx.2020.04.044

For peer review only

10 Regjeringen. Omfattende tiltak for å bekjempe koronaviruset. 2020.https://www.regjeringen.no/no/aktuelt/nye-tiltak/id2693327/ (accessed 10 Nov 2020).

11 Blendon RJ, Benson JM, DesRoches CM, *et al.* The Public's Response to Severe Acute Respiratory Syndrome in Toronto and the United States. *Clin Infect Dis* 2004;**38**:925–31. doi:10.1086/382355

12 Brooks SK, Webster RK, Smith LE, *et al.* The psychological impact of quarantine and how to reduce it: rapid review of the evidence. *The Lancet* 2020;**395**:912–20. doi:10.1016/S0140-6736(20)30460-8

13 Yoon M-K, Kim S-Y, Ko H-S, *et al.* System effectiveness of detection, brief intervention and refer to treatment for the people with post-traumatic emotional distress by MERS: a case report of community-based proactive intervention in South Korea. *Int J Ment Health Syst* 2016;**10**:51. doi:10.1186/s13033-016-0083-5

14 Hetkamp M. Sleep disturbances, fear, and generalized anxiety during the COVID-19 shut down phase in Germany: relation to infection rates, deaths, and German stock index DAX. *Sleep Med* 2020;:4.

15 Yu H, Li M, Li Z, *et al.* Coping style, social support and psychological distress in the general Chinese population in the early stages of the COVID-19 epidemic. *BMC Psychiatry* 2020;**20**:426. doi:10.1186/s12888-020-02826-3

16 Fisher A, Roberts A, McKinlay AR, *et al.* The impact of the COVID-19 pandemic on mental health and well-being of people living with a long-term physical health condition: a qualitative study. Public and Global Health 2020. doi:10.1101/2020.12.03.20243246

17 Marcovecchio ML, Chiarelli F. The effects of acute and chronic stress on diabetes control. *Sci Signal* 2012;**5**:pt10. doi:10.1126/scisignal.2003508

18 Ikesu R, Miyawaki A, Sugiyama T, *et al.* Trends in Diabetes Care during the COVID-19 Outbreak in Japan: an Observational Study. *J Gen Intern Med* Published Online First: 19 January 2021. doi:10.1007/s11606-020-06413-w

19 Pal R, Yadav U, Verma A, *et al.* Awareness regarding COVID-19 and problems being faced by young adults with type 1 diabetes mellitus amid nationwide lockdown in India: A qualitative interview study. *Prim Care Diabetes* 2021;**15**:10–5. doi:10.1016/j.pcd.2020.07.001

20 Shi C, Zhu H, Liu J, *et al.* Barriers to Self-Management of Type 2 Diabetes During COVID-19 Medical Isolation: A Qualitative Study. *Diabetes Metab Syndr Obes Targets Ther* 2020;**Volume 13**:3713–25. doi:10.2147/DMSO.S268481

21 Verma A, Rajput R, Verma S, *et al.* Impact of lockdown in COVID 19 on glycemic control in patients with type 1 Diabetes Mellitus. *Diabetes Metab Syndr Clin Res Rev* 2020;**14**:1213–6. doi:10.1016/j.dsx.2020.07.016

22 Barchetta I, Cimini FA, Bertoccini L, *et al.* Effects of work status changes and perceived stress on glycaemic control in individuals with type 1 diabetes during COVID-19 lockdown in Italy. *Diabetes Res Clin Pract* 2020;**170**:108513. doi:10.1016/j.diabres.2020.108513

23 Aragona M, Rodia C, Bertolotto A, *et al.* Type 1 diabetes and COVID-19: The "lockdown effect." *Diabetes Res Clin Pract* 2020;**170**. doi:10.1016/j.diabres.2020.108468

For peer review only

24 Potier L, Hansel B, Larger E, *et al.* Stay-at-Home Orders During the COVID-19 Pandemic, an Opportunity to Improve Glucose Control Through Behavioral Changes in Type 1 Diabetes. *Diabetes Care* 2021;**44**:839–43. doi:10.2337/dc20-2019

25 Fernández E, Cortazar A, Bellido V. Impact of COVID-19 lockdown on glycemic control in patients with type 1 diabetes. *Diabetes Res Clin Pract* 2020;**166**:108348. doi:10.1016/j.diabres.2020.108348

26 Joensen LE, Madsen KP, Holm L, *et al.* Diabetes and COVID-19: psychosocial consequences of the COVID-19 pandemic in people with diabetes in Denmark—what characterizes people with high levels of COVID-19-related worries? *Diabet Med* 2020;**37**:1146–54. doi:10.1111/dme.14319

27 Forde R, Arente L, Ausili D, *et al.* The impact of the COVID-19 pandemic on people with diabetes and diabetes services: A pan-European survey of diabetes specialist nurses undertaken by the Foundation of European Nurses in Diabetes survey consortium. *Diabet Med* Published Online First: 30 December 2020. doi:10.1111/dme.14498

28 Grabowski D, Overgaard M, Meldgaard J, *et al.* Disrupted Self-Management and Adaption to New Diabetes Routines: A Qualitative Study of How People with Diabetes Managed Their Illness during the COVID-19 Lockdown. *Diabetology* 2021;**2**:1–15. doi:10.3390/diabetology2010001

29 Flanagan JC. The critical incident technique. *Psychol Bull* 1954;**51**:327–58. doi:10.1037/h0061470

30 Fridlund B, Henricson, M., Mårtensson J. Critical Incident Technique applied in nursing and healthcare sciences. *SOJ Nurs Health Care* 2017;**3**:1–5. doi:10.15226/2471-6529/3/1/00125

31 Kemppainen JK. The critical incident technique and nursing care quality research. *J Adv Nurs* 2000;**32**:1264–71. doi:10.1046/j.1365-2648.2000.01597.x

32 Magliah SF, Zarif HA, Althubaiti A, *et al.* Managing Type 1 Diabetes among Saudi adults on insulin pump therapy during the COVID-19 lockdown. *Diabetes Metab Syndr Clin Res Rev* 2021;**15**:63–8. doi:10.1016/j.dsx.2020.12.013

33 Barone MTU, Harnik SB, de Luca PV, *et al.* The impact of COVID-19 on people with diabetes in Brazil. *Diabetes Res Clin Pract* 2020;**166**:108304. doi:10.1016/j.diabres.2020.108304

34 Di Dalmazi G, Maltoni G, Bongiorno C, *et al.* Comparison of the effects of lockdown due to COVID-19 on glucose patterns among children, adolescents, and adults with type 1 diabetes: CGM study. *BMJ Open Diabetes Res Care* 2020;**8**:e001664. doi:10.1136/bmjdrc-2020-001664

35 Tornese G, Ceconi V, Monasta L, *et al.* Glycemic Control in Type 1 Diabetes Mellitus During COVID-19 Quarantine and the Role of In-Home Physical Activity. *Diabetes Technol Ther* 2020;**22**:462–7. doi:10.1089/dia.2020.0169

36 Christoforidis A, Kavoura E, Nemtsa A, *et al.* Coronavirus lockdown effect on type 1 diabetes management on children wearing insulin pump equipped with continuous glucose monitoring system. *Diabetes Res Clin Pract* 2020;**166**:108307. doi:10.1016/j.diabres.2020.108307

37 Capaldo B, Annuzzi G, Creanza A, *et al.* Blood Glucose Control During Lockdown for COVID-19: CGM Metrics in Italian Adults With Type 1 Diabetes. *Diabetes Care* 2020;**43**:e88–9. doi:10.2337/dc20-1127

For peer review only

38 Maddaloni E, Coraggio L, Pieralice S, *et al.* Effects of COVID-19 Lockdown on Glucose Control: Continuous Glucose Monitoring Data From People With Diabetes on Intensive Insulin Therapy. *Diabetes Care* 2020;**43**:e86–7. doi:10.2337/dc20-0954

39 Fisher L, Hessler D, Polonsky W, *et al.* Diabetes distress in adults with type 1 diabetes: Prevalence, incidence and change over time. *J Diabetes Complications* 2016;**30**:1123–8. doi:10.1016/j.jdiacomp.2016.03.032

40 Freeston M, Tiplady A, Mawn L, *et al.* Towards a model of uncertainty distress in the context of Coronavirus (COVID-19). *Cogn Behav Ther* 2020;**13**:e31. doi:10.1017/S1754470X2000029X

41 Lindstrom B. Contextualizing salutogenesis and Antonovsky in public health development. *Health Promot Int* 2006;**21**:238–44. doi:10.1093/heapro/dal016

42 Antonovsky A, Sjøbu A. *Helsens mysterium: den salutogene modellen*. Oslo: : Gyldendal akademisk 2013.

43 Bauer GF, Eriksson M, Espnes GA, *et al.*, editors. *The Handbook of Salutogenesis*. 1st ed. 2017. Cham: : Springer International Publishing : Imprint: Springer 2017. doi:10.1007/978-3-319-04600-6

44 Raknes G, Strøm MS, Sulo G, *et al.* Lockdown and non-COVID-19 deaths: Cause-specific mortality during the first wave of the 2020 pandemic in Norway. A population-based register study. Epidemiology 2021. doi:10.1101/2021.02.09.21251326

45 Polit DF, Beck CT. *Nursing research: generating and assessing evidence for nursing practice*. Tenth edition. Philadelphia: : Wolters Kluwer Health 2017.

46 Malterud K. Qualitative research: standards, challenges, and guidelines. *The Lancet* 2001;**358**:483–8. doi:10.1016/S0140-6736(01)05627-6

47 Knox S, Burkard AW. Qualitative research interviews. *Psychother Res* 2009;**19**:566–75. doi:10.1080/10503300802702105

48 Malterud K. *Kvalitative forskningsmetoder for medisin og helsefag: en innføring*. Oslo: : Universitetsforlaget 2017.

49 Weekly epidemiological update on COVID-19 - 8 June 2021. https://www.who.int/publications/m/item/weekly-epidemiological-update-on-covid-19---8-june-2021 (accessed 12 Jun 2021).

50 Norwegian Institute of Public Health, (NIPH). Statistics coronavirus and covid-19 - Key figures for Norway. 2021.https://www.fhi.no/sv/smittsomme-sykdommer/corona/dags--og-ukerapporter/dags--og-ukerapporter-om-koronavirus/ (accessed 28 Apr 2021).

For peer review only

Table 1 Description of the participants of the study and characteristics of the interview situation.

Person	*Age range*	*DM Duration years*	*A1c mmol/mol*	*Diabetes complications*	*COVID-19*[1]	*Occupation exposed*	*Living alone*	*Interview form*	*Interview duration minutes*	*Site*[2]
1	60s	46	61	yes			no	meeting	56	1
2	40s	23	64	yes			no	video	25	1
3	30s	23	51	yes			yes	meeting	51	2
4	50s	45	66	yes		Kindergarten	yes	video	39	2
5	20s	17	60	no			no	video	17	2
6	40s	3	63	no	(+)		no	video	33	2
7	50s	19	54	no			no	video	59	1
8	20s	25	50	no			no	video	43	3
9	50s	21	51	no	+		no	video	38	3
10	50s	14	66	no	+		no	video	42	3
11	50s	9	63	no			no	video	22	3
12	40s	29	53	yes		School assistant	no	video	42	3
13	30s	20	65	no			yes	telephone	57	3
14	20s	10	46	no		Healthcare	yes	video	27	3
15	50s	53	57	yes			no	video	35	2
16	40s	44	78	no			no	telephone	45	2
17	40s	37	53	no		Teacher	no	video	36	2
18	50s	32	48	yes			no	telephone	93	2
19	50s	26	53	no			yes	video	51	3
Mean	**45 years**	**26 years**	**58 mmol/mol**						**43 min**	

[1] The symbol + denotes a confirmed case of COVID-19 infection, and the symbol (+) denotes suspected but unconfirmed infection.

[2] The three different sites of the study are represented by numbers 1, 2 and 3. The total patient populations at the three hospitals were 160 000 (1), 240 000 (2) and 560 000 (3). According to the annual report from the Norwegian Diabetes Register for Adults, 393 (1), 699 (2) and 1230 (3) patients with type 1 diabetes are treated and followed up at these outpatient clinics.

Table 2 Interview guide for the study, including probing questions and background information.

TOPIC	Q1	Q2	Q3	Q4	Q5	Q6	**Probing questions**	**Background information**
**Topic1: COVID-19 and daily life and coping with the situation**	When you first heard about the COVID-19 virus and that it had spread to Norway, what were your thoughts?	Do you have any thoughts about whether this virus is dangerous for you, and if so, how have these thoughts affected you?	Have you taken any specific actions to take care of your diabetes treatment during this period?	Can you describe how your everyday life is or was affected by the COVID-19 pandemic?	Can you describe what it was like for you and your family when society started reopening?	Can you describe what it has been like for you to take care of the diabetes treatment during this time?	Where did this happen?<br><br>What did you do in this situation / can you explain more about what happened?<br><br>What did you think in this situation?<br><br>What feelings did you have in this situation?<br><br>What do you think was the most demanding part of this situation?<br><br>What does this mean for you in the future?	For how long have you had diabetes?<br><br>Have you developed any diabetes complications?<br><br>What was your last registered A1c?
**Topic 2: Sources of information about COVID-19 and diabetes**	How did you and your family relate to news and media coverage of the pandemic?	Can you describe how you got information about the COVID-19 pandemic?						
**Topic 3: COVID-19 and help from the healthcare service**	What expectations have you had about possible help / support from the health service to cope with the situation?	Do you feel that your expectations have been met?	If there was a new pandemic, what kind of health service would you wish to be established for people in risk groups?					

For peer review only

Table 3 Data analysis of experiences from quotations, grouped by subcategory, category and main area.

QUOTATION	SUBCATEGORY	CATEGORY	MAIN AREA
"Because it was so uncertain whether I was more at risk or, uhm yes, or how things were because no one knew that much. So, I thought it was scary." (3)	Uncertainty caused by belonging to a high-risk group	Concerns related to COVID-19 and T1DM	Increased psychosocial burden of T1DM during the COVID-19 pandemic
"But I was genuinely afraid of getting this, because I thought that if I get it, I'll die." (9)	Anxiety concerning infection		
"The worst thing about the corona, when it came, it was all the uncertainty – we knew nothing." (18)	Concerns about the overall situation		
"I paid attention at first, and then I thought this is not good, I will get depressed from seeing this, all this intimidating propaganda." (19)	Continuous media focus raised concerns	A need for knowledge about T1DM and COVID-19	
"If I've been missing anything to some extent, it might getting insight into why diabetics are particularly vulnerable in a corona situation." (10)	Need for balanced information for people in risk groups		
"I contacted the doctor's office. I also contacted the hospital to find out if I was at risk and what to do if called back to work." (4)	A desire for personalized information		
"No, it's more the social discomfort you feel when people you meet sort of go for a hug and then you have to say 'You know what? I don't do that'." (14)	Challenges in social distancing	Consequences in social relations from COVID-19-related restrictions	
"And it was – especially for the children – it was probably the most demanding, that they had to avoid being with others for such a long time." (10)	Increased restrictions for family members of people with T1DM		
"So, it was kind of that I should not return to work, so that they [her colleagues] would not get a bad conscience if I were to die." (4)	Restrictions related to being in a high-risk group due to T1DM		
"I felt, when we locked down, that … that the start was completely awful because it, uhm, it completely changed my routines." (3)	Difficulties creating routines in everyday life at home	Altered routines due to COVID-19 affected T1DM self-management	Changed conditions for T1DM treatment during the COVID-19 pandemic

For peer review only - http://bmjopen.bmj.com/site/about/guidelines.xhtml

"Uhm yes, but it makes one stay indoors more and such, and then it's easier to make a little extra-good food so, …" (2)	Difficulties maintaining a healthy lifestyle	
"Surprisingly, the treatment has been better than ever before. But it has to do with the fact that I got a new type of insulin pump. And it has really made everyday life a lot easier." (8)	The benefit of glucose sensor and insulin pump in diabetes treatment	
"I had an incredibly high fever and had an insanely high blood glucose." (9)	Challenging fluctuations in blood glucose during COVID-19 disease	
"It [the blood glucose] has been affected because I, well it's much busier at work [teacher] because we wash desks and surfaces all the time." (17)	New routines at work to prevent the spread of COVID-19	Changes in the work or study situation due to COVID-19 affecting T1DM self-management
"But clearly, the lack of breaks and not being able to eat and such, I must say that I have sometimes felt that it has been tough at work." (17)	Increased workload due to COVID-19 caused stress	
"All the time I feel that I have kind of a 'sting' in the back of my mind, whether I might be laid off again." (18)	Financial uncertainty caused stress	
"It's even easier, when I work from home, to maintain good blood glucose control, compared with running around taking those measurements at work all the time." (10)	More predictable everyday life gave more time to focus on T1DM self-management	
"The one [health check] before the summer was just completely eliminated, so there was a year in between check-ups and I don't think that is good." (13)	Lack of or delayed follow-up from the specialist health service	Reorganization of the health service due to COVID-19 affecting T1DM treatment
"I must admit that in the beginning I thought 'This is a state of emergency, now I have to stock up on insulin'." (15)	Concerns regarding access to medicines	
"And if I should end up with someone who does not necessarily have full control over what blood glucose is all about in such an intensive ward, then yes. (7)	Concerns about ICU healthcare professionals' knowledge of specialised diabetes treatment in case of severe illness and need for help	
"In the end I called the diabetes nurse and asked: 'What am I going to do? I can't get my blood glucose down'." (9)	Availability of healthcare professionals by telephone	

Table 4 Data analysis of actions from quotations, to subcategories, via categories up to the main areas.

QUOTATION	SUBCATEGORY	CATEGORY	MAIN AREA
"I was very, very conscious about keeping my distance to others, and as I said, I wore a mask as well." (18)	Increased awareness of complying with infection control advice	Practical strategies to avoid being infected with COVID-19	Actions to handle psychosocial strain related to T1DM and COVID-19
"Every Saturday he [her brother] goes to the store and buys groceries for me." (15)	Getting help from others to obtain food and medicine		
"We can't let you [the children] take the bus unnecessarily, you should not go to a party where there are 50 people, that is not what we should do now. So yes, they must consider me." (16)	Limiting family members' number of social contacts		
"I have a good friend who has bone marrow cancer. And we both agreed to it because she is very restrictive about who she socializes with too, so the two of us had a lot of contact." (4)	Choosing a few people to meet and socialize with	Measures to maintain social contact with others	
"And in this hobby community they are good at organizing such online meetings, so we sit at our PCs together instead of alone." (12)	Creating alternative arenas for socialization		
"I'm so fortunate that I have a husband that I live with. I'm married, you know, so I think that the loneliness that many feel. I'm much better off." (15)	Finding joy and support within the family		
"And then I thought well, it's not certain that it's really bad that I get it [COVID-19] because I'm pretty healthy, I have a good Hba1c, my immune system is kind of good." (5)	Positive cognitive reflections to deal with the situation	Mental strategies to deal with the situation	
"I kind of tried to avoid maybe the newspapers as much as possible, because I feel that … well, it affects me in a negative sense." (3)	Selective attitude towards media information		
"I asked my doctor what should I do? Should I just continue working from home and answer technical questions, or should I travel and work as normal?" (19)	Actively searching for personalized information		

"I quickly decided that I must live as normally as possible. Eat as normal. Be physically active outside. Keep in touch with others in a safe way." (4)	Creating predictable everyday routines for T1DM self-management	Creating a safe structure in daily life	Actions to handle changed conditions for T1DM treatment during the COVID-19 pandemic
"So then I eat in the car- instead of going in there to eat with the others [on a construction site]." (2)	Starting new routines at work for infection control		
"I've been pretending that it's a normal day. I get up at regular times, shower, put on make-up, etc." (15)	Creating appropriate home-office routines		
"And I have enough things to do so that there will not be much effort spent on thinking too much, to put it that way." (11)	Consciously finding meaningful pursuits to avoid brooding		
"And also I think we are very fortunate to have the diabetes system we have here in Norway; that I was allowed to get Freestyle Libre and such." (10)	Using medical technical equipment actively in treating diabetes	Measures to ensure proper T1DM self-management during the COVID-19 pandemic	
"I could not go to the gym, so I had to use nature instead." (4)	Alternative physical activities to maintain a healthy lifestyle during the COVID-19 pandemic		
"And tried to inject, took higher and higher insulin doses." (9)	Titrate insulin doses in relation to blood glucose fluctuations during COVID-19 disease		
"Then I was isolated in the bedroom by my wife. I was not allowed to go out." (9)	Isolating due to COVID-19 illness		
"And about medicine and stuff. I have always had a stock of insulin." (4)	Keeping a stockpile of insulin available for emergencies		

COREQ (COnsolidated criteria for REporting Qualitative research) Checklist

A checklist of items that should be included in reports of qualitative research. You must report the page number in your manuscript where you consider each of the items listed in this checklist. If you have not included this information, either revise your manuscript accordingly before submitting or note N/A.

Topic	Item No.	Guide Questions/Description	Reported on Page No.
**Domain 1: Research team and reflexivity**			
Personal characteristics			
Interviewer/facilitator	1	Which author/s conducted the interview or focus group?	
Credentials	2	What were the researcher's credentials? E.g. PhD, MD	
Occupation	3	What was their occupation at the time of the study?	
Gender	4	Was the researcher male or female?	
Experience and training	5	What experience or training did the researcher have?	
Relationship with participants			
Relationship established	6	Was a relationship established prior to study commencement?	
Participant knowledge of the interviewer	7	What did the participants know about the researcher? e.g. personal goals, reasons for doing the research	
Interviewer characteristics	8	What characteristics were reported about the inter viewer/facilitator? e.g. Bias, assumptions, reasons and interests in the research topic	
**Domain 2: Study design**			
Theoretical framework			
Methodological orientation and Theory	9	What methodological orientation was stated to underpin the study? e.g. grounded theory, discourse analysis, ethnography, phenomenology, content analysis	
Participant selection			
Sampling	10	How were participants selected? e.g. purposive, convenience, consecutive, snowball	
Method of approach	11	How were participants approached? e.g. face-to-face, telephone, mail, email	
Sample size	12	How many participants were in the study?	
Non-participation	13	How many people refused to participate or dropped out? Reasons?	
Setting			
Setting of data collection	14	Where was the data collected? e.g. home, clinic, workplace	
Presence of non-participants	15	Was anyone else present besides the participants and researchers?	
Description of sample	16	What are the important characteristics of the sample? e.g. demographic data, date	
Data collection			
Interview guide	17	Were questions, prompts, guides provided by the authors? Was it pilot tested?	
Repeat interviews	18	Were repeat inter views carried out? If yes, how many?	
Audio/visual recording	19	Did the research use audio or visual recording to collect the data?	
Field notes	20	Were field notes made during and/or after the inter view or focus group?	
Duration	21	What was the duration of the inter views or focus group?	
Data saturation	22	Was data saturation discussed?	
Transcripts returned	23	Were transcripts returned to participants for comment and/or	

For peer review only

Topic	Item No.	Guide Questions/Description	Reported on Page No.
		correction?	
**Domain 3: analysis and findings**			
Data analysis			
Number of data coders	24	How many data coders coded the data?	
Description of the coding tree	25	Did authors provide a description of the coding tree?	
Derivation of themes	26	Were themes identified in advance or derived from the data?	
Software	27	What software, if applicable, was used to manage the data?	
Participant checking	28	Did participants provide feedback on the findings?	
Reporting			
Quotations presented	29	Were participant quotations presented to illustrate the themes/findings? Was each quotation identified? e.g. participant number	
Data and findings consistent	30	Was there consistency between the data presented and the findings?	
Clarity of major themes	31	Were major themes clearly presented in the findings?	
Clarity of minor themes	32	Is there a description of diverse cases or discussion of minor themes?	

Developed from: Tong A, Sainsbury P, Craig J. Consolidated criteria for reporting qualitative research (COREQ): a 32-item checklist for interviews and focus groups. *International Journal for Quality in Health Care*. 2007. Volume 19, Number 6: pp. 349 – 357

Once you have completed this checklist, please save a copy and upload it as part of your submission. DO NOT include this checklist as part of the main manuscript document. It must be uploaded as a separate file.

For peer review only

BMJ Open

Experiences and actions related to living with type 1 diabetes during the COVID-19 pandemic in Norway - A qualitative study conducted during July - December 2020

Journal:	*BMJ Open*
Manuscript ID	bmjopen-2021-056027.R2
Article Type:	Original research
Date Submitted by the Author:	03-Mar-2022
Complete List of Authors:	Pleym, Karin; Western Norway University of Applied Sciences, Department of Health and Caring Sciences; Vestre Viken Hospital Trust, Department of Medicine<br>Iversen, Marjolein; Western Norway University of Applied Sciences, Department of Health and Caring Sciences<br>Broström, Anders; Jönköping University, Department of Nursing, School of Health and Welfare; Linköping University Hospital, Department of Clinical Neurophysiology
**Primary Subject Heading**:	Diabetes and endocrinology
Secondary Subject Heading:	Nursing, Qualitative research
Keywords:	COVID-19, QUALITATIVE RESEARCH, DIABETES & ENDOCRINOLOGY

SCHOLARONE™
Manuscripts

I, the Submitting Author has the right to grant and does grant on behalf of all authors of the Work (as defined in the below author licence), an exclusive licence and/or a non-exclusive licence for contributions from authors who are: i) UK Crown employees; ii) where BMJ has agreed a CC-BY licence shall apply, and/or iii) in accordance with the terms applicable for US Federal Government officers or employees acting as part of their official duties; on a worldwide, perpetual, irrevocable, royalty-free basis to BMJ Publishing Group Ltd ("BMJ") its licensees and where the relevant Journal is co-owned by BMJ to the co-owners of the Journal, to publish the Work in this journal and any other BMJ products and to exploit all rights, as set out in our licence.

The Submitting Author accepts and understands that any supply made under these terms is made by BMJ to the Submitting Author unless you are acting as an employee on behalf of your employer or a postgraduate student of an affiliated institution which is paying any applicable article publishing charge ("APC") for Open Access articles. Where the Submitting Author wishes to make the Work available on an Open Access basis (and intends to pay the relevant APC), the terms of reuse of such Open Access shall be governed by a Creative Commons licence – details of these licences and which Creative Commons licence will apply to this Work are set out in our licence referred to above.

Other than as permitted in any relevant BMJ Author's Self Archiving Policies, I confirm this Work has not been accepted for publication elsewhere, is not being considered for publication elsewhere and does not duplicate material already published. I confirm all authors consent to publication of this Work and authorise the granting of this licence.

For peer review only

Experiences and actions related to living with type 1 diabetes during the COVID-19 pandemic in Norway - A qualitative study conducted during July - December 2020

Karin Pleym RN, Msc[1,2], Marjolein M. Iversen PhD[1] and Anders Broström PhD[3,4]

1. Faculty of Health and Social Science, Western University of Applied Sciences, Norway

2. Department of Medicine, Vestre Viken Drammen Hospital, Norway

3. Department of Nursing, School of Health and Welfare, Jönköping University, Jönköping, Sweden

4. Department of Clinical Neurophysiology, Linköping University Hospital, Linköping, Sweden

Corresponding author: Marjolein M. Iversen, Inndalsveien 28, 5063 Bergen, Norway. Tel. +47 902 55 044, e-mail: Marjolein.Memelink.Iversen@hvl.no

For peer review only

Word count:

Abstract: 298 words

Manuscript: 4160

Tables: 4

Figures: 0

References: 50

Abstract

Objectives: The aim of this study was to describe the experiences of adults with type 1 diabetes (T1DM) during the COVID-19 pandemic in Norway, and what actions they took to cope with the situation.

Design: An inductive, descriptive design applying the critical incident technique (CIT) was used to collect qualitative data between July 2020 and December 2020.

Setting: A strategic selection was made from diabetes specialist outpatient clinics at three different hospitals in eastern Norway. The hospitals, two community hospitals and one university hospital, were situated in both rural and urban areas.

Participants: Inclusion criteria were people with T1DM aged 18–65 years. Exclusion criteria were pregnancy, people with chronic pulmonary disorders, people with active cancer diseases and people diagnosed with a myocardial infarction or stroke during the previous six months. Semi-structured individual interviews with 19 people with T1DM were conducted.

Results: Experiences were categorized into two main areas: "Increased psychosocial burden of T1DM during the COVID-19 pandemic" and "Changed conditions for T1DM treatment during the COVID-19 pandemic". Uncertainty distress and social consequences from infection control measures contributed to the burden of T1DM. Disrupted T1DM follow-up and altered daily routines created challenges. However, having increased time to focus on T1DM self-management during lockdown represented an improvement. Actions to handle the situation were categorized into two main areas: "Actions to handle psychosocial strain related to T1DM and COVID-19" and "Actions to handle changed conditions for T1DM treatment during the COVID-19 pandemic".

Conclusions: Patients experienced an increased psychosocial burden of T1DM and difficulties from a disrupted daily life affecting T1DM self-management routines. Uncertainty-reducing behaviours and actions to adapt to the situation provided a general sense of coping despite these difficulties. Tailored information and follow-up by telephone or video-call was emphasized to reduce uncertainly distress and support adequate diabetes T1DM self-management.

For peer review only

Strengths and limitations of this study

- Three researchers with good knowledge of both diabetes and critical incident technique have been involved throughout the study.
- Utilising the critical incident technique, we were able to focus on both experiences and actions used by people with type 1 diabetes during the COVID-19 pandemic in Norway.
- The majority of the interviews were performed by video-call giving the participants the comfort of familiar environments and contributing to a safe interview situation.
- A possible paradox in the study may be that subjects who suffer from lock-down, usually could be the ones who want to participate in the study.
- Norway had an early complete lock-down prior to the data collection, and relatively few deaths from COVID-19 compared to other European countries.

For peer review only

INTRODUCTION

The outbreak of SARS-CoV-2-induced coronavirus disease 2019 (COVID-19) was declared a global pandemic by the World Health Organization on 11 March 2020.[1] The COVID-19 disease ranges in severity from asymptomatic or mild cases to severe illness and death.[2,3] Diabetes has been identified as one of several underlying health conditions associated with increased severity and higher mortality rates from COVID-19 illness.[4–9]

In Norway the government implemented strict interventions to limit the spread of the virus.[10] The infection control measures had significant impacts on the population's everyday life. Previous reports from preceding coronavirus epidemics such as SARS-CoV (SARS) in 2002–2003 and the Middle East Respiratory Syndrome coronavirus (MERS) in 2012–2015 showed evidence of increased psychosocial and mental health challenges.[11–13]

Similarly, psychosocial challenges caused by the COVID-19 pandemic have been seen in the general population.[14–16] The massive media coverage and the general perception of the severity of the situation had potential to cause stress and anxiety. People with type 1 diabetes (T1DM) can be more vulnerable to stress in terms of a direct effect of stress hormones on blood glucose levels and an indirect effect on the person's behaviour related to diabetes self-management.[17] Previous studies have shown that people with T1DM experienced disrupted follow-up from specialist outpatient clinics during the COVID-19 pandemic and challenges affecting T1DM self-management routines.[18–22] Other studies, however, showed improved glycaemic control in periods of lockdown of society.[23–25] A cross-sectional survey from Denmark, in which 2 430 adults with diabetes completed an online questionnaire, indicated that people with diabetes have COVID-19-specific worries about their diabetes associated with poorer psychosocial health.[26] In line with these results, diabetes nurses across Europe have reported a significant increase in physical and psychological problems in patients with diabetes.[27] A qualitative study has provided insight into the experiences of people diagnosed with various types of diabetes in the initial phase of lockdown in Denmark.[28] However, little is known about how people with T1DM handled daily life during the pandemic over a longer period of time that includes the reopening of society. Knowledge of the experiences and actions of people with T1DM during

For peer review only

the COVID-19 pandemic may provide insight into strategies for handling the situation as well as possible needs for assistance from the healthcare system. This knowledge may in turn be valuable for other risk groups and in the event of similar situations in future. The aim of this study was to describe the experiences of adults with T1DM during the COVID-19 pandemic in Norway and what actions they took to cope with their situation.

RESEARCH DESIGN AND METHODS

For peer review only

Design and method description

This qualitative study used an inductive, descriptive design, applying the critical incident technique (CIT).[29] A “critical incident” represents a major event of great importance to the person involved, and is an essential part of the CIT methodology. Critical incidents consist of positive or negative experiences in addition to actions carried out to handle the phenomenon in question.[30] Due to its flexibility and the focus on the patient perspective, CIT is considered an eligible method for use in healthcare research.[31] The method enables informants to describe significant incidents as well as their experiences and actions. Consequently, CIT is a suitable method for exploring the research question of this study.

Ethical considerations

The Regional Committee for Medical and Health Research Ethics in South East Norway (REC-154546), the Norwegian Centre for Research Data (NSD) (NSD-39284), the local data protection officers at the three study sites and Western Norway University of Applied Science evaluated the study protocol and accepted it before study start.

Participants and setting

The informants were recruited from the diabetes specialist outpatient clinics at three different hospitals in eastern Norway. The hospitals, two community hospitals and one university hospital, were situated in both rural and urban areas. The total patient

populations at the hospitals were 160 000, 240 000 and 560 000 respectively. People with T1DM will typically meet with a diabetes nurse or an endocrinologist in the diabetes outpatient clinics every three to six months.

A strategic selection was made to ensure a heterogeneous group of informants. Inclusion criteria were people with T1DM aged 18–65 years. Exclusion criteria were pregnancy, people with chronic pulmonary disorders, people with active cancer diseases and people diagnosed with a myocardial infarction or stroke during the previous six months. Characteristics such as gender, age, T1DM duration, metabolic control and having an occupation with increased exposure to possible infection were used in the selection of informants (Table 1). Based on the criteria for strategic selection, a local designated diabetes nurse provided oral information about the study to potential participants. When interest was expressed, the nurse asked for oral consent to be contacted by the first author by telephone to provide further information about the study. The first author then made contact by telephone and gave additional information, and written information was sent to the potential participant by mail. After written informed consent was returned to the first author, an appointment for an interview was made. Three informants later cancelled the interview appointment due to a busy schedule (2) or illness (1). In total, 19 people with T1DM were included in the study.

-Insert Table 1

Data collection

Descriptions of experiences and actions were obtained through semi-structured individual interviews. Due to the ongoing pandemic, informants could choose to conduct the interviews in person, by video call or by telephone. The video interviews were conducted on an encrypted web platform.

A semi-structured interview was performed based on an interview guide. The interview guide was developed by all authors together, who have extensive clinical and methodological competence. A user representative from the local diabetes association reviewed the interview guide and gave advice on the content. After three interviews were conducted, the transcriptions were discussed within the research team. The interview guide consisted of three main topics and probing questions as presented in Table 2.

For peer review only

The interviews were conducted between July 2020 and December 2020. Norway was in complete lockdown from March 12th to mid-April 2020. After this, a cautious reopening of society was initiated. Nevertheless, the authorities' advice on social distancing and hygiene measures was maintained. In July 2020, when the first interviews were conducted, the community was relatively open. Later in the autumn, infection rates started to increase again, which led to a stronger focus on measures such as working from home, partially closed schools, the obligation to wear masks and so forth. There was no current Sars-Cov2 vaccine available in Norway at the time of this study.

The interviews were all performed by the first author and recorded on a digital recorder. The first author transcribed 12 and a project assistant transcribed seven of the audio recordings verbatim.

-Insert Table 2

Patient and Public Involvement

A user representative from the local Diabetes Association gave input in the preparation of the interview guide. Patients and the public were not involved in the development of the research question, design and conduct of this study. However, the results of the study will be disseminated on a local and/or national meeting in cooperation with the Diabetes Association.

Data analysis

The study was analysed in line with CIT as described by Fridlund et al.[30] Data were regularly discussed in the research team until consensus regarding categorization was achieved among the three authors. The first author read the interviews several times to obtain an overall picture of the content. Experiences and actions related to the purpose of the study were highlighted in the text and delimited. A total of 313 experiences and 185 actions was identified and categorized. Delimitations from the text were then compared for similarities in an effort to group them into subcategories. When the subcategories were identified, a new process of exploration and comparison was performed to group the subcategories into categories. The purpose of a category is to describe the general character of the subcategories. Following the establishment of

For peer review only

categories, a new effort was made to group categories into main areas. The purpose of constructing main areas was to describe the overall theme of the data. Descriptions of experiences and actions were analysed separately. This process led to the finding of 21 subcategories of experiences and 18 subcategories of actions. The subcategories were then structured into six categories of experiences and five categories of actions. Finally, two main areas of experiences and actions were identified.

RESULTS

In total, 10 female and nine male people with T1DM participated in the study, of which two had been diagnosed with COVID-19. All the informants were Norwegian born with the same cultural background (Table 1).

Experiences

Table 3 presents the experiences of people with T1DM during the COVID-19 pandemic in Norway, including direct quotations from the interviews. Two main areas emerged from the data analysis of experiences: “Increased psychosocial burden of T1DM during the COVID-19 pandemic” and “Changed conditions for T1DM treatment during the COVID-19 pandemic”.

-Insert Table 3

Increased psychosocial burden of T1DM during the COVID-19 pandemic

Concerns related to COVID-19 and T1DM

Informants described uncertainty caused by belonging to a risk group. This included fear of getting sick from COVID-19 and confusion about the reasons why T1DM was associated with higher risk of a fatal outcome. General anxiety about the uncertainty of the overall situation was described as difficult.

For peer review only

A need for knowledge about T1DM and COVID-19

The continuous media coverage caused anxiety and worries. The informants expressed a need for balanced information aimed at people in risk groups. Additionally, personalized information was desired and sought by contacting familiar healthcare providers such as the general practitioner or the diabetes nurse.

Consequences for social relations as a result of COVID-19-related restrictions

This category describes the various difficulties the informants experienced while socializing with family, friends, colleagues and others. To avoid bringing the COVID-19 virus into the home, family members of people with T1DM were subjected to stricter restrictions than their peers. For instance, the children of one informant were not allowed to use public transport, and the son of a divorced woman moved temporarily to live with his father. Experiences of social limitations in the workplace due to the T1DM diagnosis were reported. For example, one of the informants was told that it was not desirable for her to return to the workplace after the termination of lockdown, as colleagues were afraid of infecting her.

Changed conditions for T1DM treatment during the COVID-19 pandemic

Altered routines due to COVID-19 affecting T1DM self-management

Restrictions implemented by the government to prevent the spread of COVID-19 created changes in daily routines which in turn affected T1DM self-management. Informants expressed difficulties in maintaining a healthy lifestyle in terms of physical activity and healthy diet. To have a good time within the family, food such as cakes and ice cream were served more frequently. Since the gyms were closed, less familiar activities were initiated which made it more difficult to predict correct insulin doses. Informants who contracted COVID-19 experienced large fluctuations in and high levels of blood glucose during the period of illness. Having access to continuous blood glucose monitoring (CGM) and insulin pumps was seen as an advantage in handling this situation. Reaching out to the diabetes nurse by telephone for advice on how to titrate insulin doses gave a sense of security.

For peer review only

Changes in the work or study situation due to COVID-19 affecting T1DM self-management

Measures to prevent infection in the workplace led to new routines. This had an impact on meal breaks, time spent being physically active and opportunities for social contact at work. Some, such as a teacher and a mail delivery worker, experienced increased work pressure and new work tasks, whereas others experienced a more predictable daily life with less stress and more time to take care of T1DM self-management. Being laid off from work because of lockdown gave rise to financial insecurity. This led to stress which in turn affected blood glucose levels.

Reorganization of the health service due to COVID-19 affecting T1DM-treatment

Informants experienced delayed follow-up from the specialist outpatient clinic. This was due to the reorganization of hospital resources to prepare for a large number of COVID-19 patients. The authorities introduced a temporary rationing of insulin to prevent hoarding and ensure access. This led to concerns about the availability of medications and created worries about the inconvenience of having to go to the pharmacy more frequently. Informants' concerns about possible lack of competence in the intensive care unit regarding specialized T1DM treatment made the risk of becoming severely ill from COVID-19 even more frightening. These concerns related to previous experiences from hospitalizations. Having experienced easy access to healthcare professionals before the pandemic made them feel confident that they could reconnect for information and advice when needed.

Actions

Table 4 presents the actions of people with T1DM during the COVID-19 pandemic in Norway, including direct quotations from the interviews. The two main areas of actions that emerged from the data analysis were "Actions to handle psychosocial strain related to T1DM and COVID-19" and "Actions to handle changed conditions for T1DM treatment during the COVID-19 pandemic". The analysis of actions is described in Table 4.

For peer review only

-Insert Table 4

Actions to handle psychosocial strain related to T1DM and COVID-19

Practical strategies to avoid being infected with COVID-19

Informants described increased awareness of complying with general advice on infection control. Measures such as social distancing and frequent hand-washing became a daily routine. Some started using a mask early on. Avoiding crowded places and receiving help from others for grocery shopping and providing necessary medications were strategies adopted to prevent infection. Limiting the number of social contacts for the family as a whole was considered necessary to avoid infection.

Measures to maintain social contact

Informants described finding support and joy within the family. Choosing a few people to meet regularly gave them the opportunity to maintain social contact outside the home. Children of parents with T1DM were allowed to choose one or two friends to play with. Both new and familiar digital platforms served as alternative arenas for social interaction.

Mental strategies to deal with the situation

Informants sought personalized information about their risk status. Positive thinking was one of the main strategies to cope with the overall situation. Furthermore, informants actively avoided media coverage of COVID-19.

Actions to handle changed conditions for T1DM treatment during the COVID-19 pandemic

Creating a safe structure in daily life

Predictable routines were created to facilitate T1DM self-management. New job routines were implemented to protect the informants from being infected. Working from home was challenging, but establishing home-office routines was experienced as a positive factor. Lack

For peer review only

of routine made handling work and T1DM self-management difficult. Meaningful activities at home were found helpful to avoid pondering about the situation.

Measures to ensure proper T1DM self- management during the COVID-19 pandemic

Medical devices such as CGM and insulin pumps were perceived as useful in the treatment of T1DM and contributed to good metabolic control. Furthermore, informants found alternative physical activities to maintain a healthy lifestyle when the fitness centres were closed. Keeping a storage of insulin led to a sense of security. For those who contracted COVID-19, high blood glucose levels caused by the infection were met with efforts to titrate insulin doses, and informants called the diabetes nurse for support and advice on this matter.

CONCLUSION

This study aimed to describe the experiences and actions of people with T1DM during the COVID-19 pandemic in Norway between July and December 2020, when no current Sars-Cov2 vaccine was available in Norway. Results indicate that the psychosocial burden of living with T1DM increased during the pandemic. Uncertainty caused by belonging to a risk group, fear of severe illness and strict adherence to social distancing for the family as a whole contributed to the burden of T1DM. Additionally, we found that infection control measures and reorganization of the healthcare system represented challenges in the form of changed conditions for T1DM treatment. Applying manageable, understandable and meaningful actions to deal with the psychosocial strain and the disrupted daily life led to a general sense of coping despite difficulties.

Alteration of daily routines brought challenges such as an increased amount of unhealthy food and less exercise. These findings are in line with previous quantitative research.[32,33] In a Saudi cross-sectional study of people with T1DM using an insulin pump, difficulties in maintaining a healthy diet and staying physically active during lockdown were seen. However, adherence to the insulin pump therapy contributed to stabilizing blood

For peer review only

glucose.[32] Participants in our study described that general stress and anxiety affected T1DM self-management in terms of fluctuations in blood glucose similar to those seen in studies from Italy.[22,34] Nevertheless, our participants also described a more predictable lifestyle in working from home that led to improved control of blood glucose levels. Similar findings of improved metabolic control have been reported from studies of children with T1DM on insulin pump therapy.[35–38] Furthermore, multiple studies that reviewed metabolic control via CGM data registered during the pandemic found improvements in both time in range and blood glucose levels,[23–25] although one study showed a tendency of increased fluctuations in physical activity and perceived stress.[34]

The finding of an increased psychosocial burden of T1DM during the COVID-19 pandemic is consistent with existing evidence from quantitative studies.[26,27] Our study provides an in-depth understanding of these psychosocial difficulties, and is consistent with a qualitative study conducted in Denmark.[28] While the Danish study focused on the experiences of people with multiple types of diabetes during lockdown in Denmark (April 2020), the results from our study focused on people with T1DM at a later stage of the pandemic (July 2020–December 2020). Therefore, we covered experiences related to the reopening of society as well as to new periods of lockdown. In comparison with the Danish study, our findings may indicate that the psychosocial burden of T1DM during the pandemic increased over time. This interpretation is supported by another qualitative study of people living with different long-term physical health conditions during the COVID-19 pandemic. In this study they found that the COVID-19 pandemic affected several aspects of mental health and well-being.[16] Furthermore, in a review of the effects of quarantine, impacts on psychosocial health were seen, and the length of the period of isolation affected the outcomes of these negative effects.[12] Even though our participants were not quarantined, they were to some extent self-isolating for extended periods, and thus the psychosocial burden might have increased accordingly.

"Diabetes distress" refers to a psychological condition associated with the burden of living with T1DM.[39] Diabetes distress might add to general stress from the severity of the pandemic situation and further increase the psychosocial burden of T1DM. In a previous study, diabetes distress was identified as one of several factors associated with poorer psychosocial health in patients with diabetes during the COVID-19 pandemic.[26]

For peer review only

Freeston et al.[40] have proposed a model of uncertainty distress in the context of the COVID-19 pandemic. According to this model, uncertainty can be defined as “the subjective negative emotions experienced in response to the as yet unknown aspects of a given situation.”[40] The degree of uncertainty distress depends on the following factors: disruption of everyday life, perceived actual threat and perceived uncertainty. In addition, individual tolerance of uncertainty influences uncertainty distress. Individual tolerance of uncertainty can be modified by uncertainty-reducing behaviours. Such behaviours may include actions such as avoidance, preparation for possible negative consequences, postponement and the search for reassurance. The actions taken by the participants in our study can be seen in the light of this model of uncertainty distress in the context of the COVID-19 pandemic. Our participants actively avoided media, they prepared for possible negative consequences by keeping a stock of insulin and by strictly complying with advices on infection control measures. Furthermore, social gatherings were postponed, and the participants searched for tailored and personalized information for reassurance.

In this study, we have described how various actions were taken by the participants to adapt, and positive thinking was one of the main strategies to cope with the overall situation. From the perspective of the theory of salutogenesis, stressors in life will not necessarily contribute to poor outcomes of health if met properly. The essential factor is that the individual finds understandable, manageable and meaningful actions to adapt. Through meaningful actions, people may achieve a sense of coherence and thus of coping despite challenging environments.[41–43]

Participants in our study emphasized the importance of access to healthcare professionals by telephone for personalized guidance. A recently published but not yet peer-reviewed study from Norway shows a surprising and significant increase in non-COVID-related deaths in patients with diabetes from March 2020 to May 2020.[44] The causal factors behind these findings are unknown, and it is not possible to draw conclusions. Nevertheless, Raknes et al. propose inappropriate avoidance of healthcare professionals and thus a lack of follow-up as a possible explanation. Based on this understanding, the importance of having healthcare professionals such as diabetes nurses available by telephone for medical advice and social support is further underlined.

For peer review only

Study limitations

We have strived to achieve trustworthiness throughout the study in terms of credibility, dependability, confirmability and transferability as described by Polit & Beck.[45] Three researchers with good knowledge of both CIT and diabetes have been involved throughout the study. The first is a diabetes specialist nurse, she has been involved in all stages of this research project. The two others are professors in nursing with extensive methodological knowledge of CIT.

The first author has clinical experience which includes answering calls from diabetes patients anxious about COVID-19. Her experience represents a factor that might threaten reflexivity. On the other hand, substantial knowledge from the field can be a valuable source of relevant and specific research.[46] The first author's clinical experience can be seen as an advantage in the process of formulating relevant questions for the interview guide. Awareness of the potential influence of previous experiences has been taken into account in the interpretation of the data. Description of the data has sought to be clear and has been discussed in depth within the research team to strengthen the credibility of the study. The interviews were all performed by the first author, which increased the likelihood that the interview guide was used in the same way in all the interviews. Out of 19 interviews, 14 were conducted by video call and three were conducted by telephone, which may have contributed to a loss of some nonverbal data.[47] On the other hand, participants had the comfort of familiar environments and did not have to worry about possible infection. We have provided a detailed description of the participants as important contextual factors regarding the dependability of the study (Table 1). In sampling, we aimed to achieve variation in gender, age, metabolic control, T1DM duration and occupation. However, we were unable to include participants with a different cultural background, which is a limitation. A possible paradox in the study may be that subjects who suffer most from lock-down, could be the ones who want to talk and seek comfort from healthcare providers. To strengthen dependability and facilitate confirmability, the systematic procedure we followed in the data analysis is clearly presented with quotations categorised and finally grouped into main areas (Table 3 and Table 4). A diversity of experiences and actions were obtained from the interviews, and this is seen as a strength when using the CIT approach.[29]

For peer review only

According to Malterud, the field and the context in which the data material is acquired will always determine the scope of the knowledge in time and space.[48] From an international perspective, Norway has had relatively few deaths from COVID-19 compared to other European countries.[49] As of the 28 April 2021 there are 753 registered deaths out of 111 685 confirmed COVID-19 cases in a population of 5 391 369 inhabitants.[50] Participants included in our study live in the eastern part of Norway close to the capital. The capital and surrounding areas are the part of Norway most affected by the COVID-19 pandemic in terms of lock-down restrictions. From this perspective, it would be fair to say that the participants in this study may have had experiences similar to people in countries with higher infection rates and deaths from COVID-19. A detailed description of the study context, the participants, method of data collection and the data analysis, including direct quotations from the interviews, are presented (Table 1, Table 2, Table 3 and Table 4) to give the reader the opportunity to determine the transferability of the findings.

Conclusion and implications for clinical practice

In conclusion, participants experienced psychosocial strain and an increased burden of T1DM. Furthermore, they described uncertainty-reducing behaviours and actions which provided a general sense of coping with handling a disrupted daily life during the COVID-19 pandemic despite difficulties.

Healthcare professionals should be aware that many people with T1DM might have lived under increased stress during the COVID-19 pandemic. Availability of healthcare professionals for personalized advice by telephone was emphasized as important to alleviate uncertainty distress, reduce diabetes distress and support adequate T1DM self-management. Access to telephone and video consultations should be implemented to ensure follow-up of people with T1DM in similar situations in future.

Further research is needed to investigate potential psychosocial impacts of the COVID-19 pandemic on people with T1DM from a family perspective. Exploring possible associations between diabetes distress, uncertainty distress and psychosocial burden was beyond the scope of this study. However, this would be an interesting topic for future research.

For peer review only - http://bmjopen.bmj.com/site/about/guidelines.xhtml

Competing interests: Karin Pleym has received for a contribution of NOK 10 000 from the regional Diabetes Association for the project.

Contribution: KP, AB and MMI designed the study. MMI facilitated data collection. KP collected the data. KP, AB and MMI contributed to data analysis KP, AB and MMI contributed to drafting the manuscript and read and approved the final manuscript.

Data availability statement: All data relevant to the study are included in the article or uploaded as supplementary information.

Acknowledgement: The authors thank the patient representative May Kvernstuen for valuable input in preparation to the interview guide. This study was partly funded by Viken County Diabetes Association.

References

1 WHO. WHO Director-General’s opening remarks at the media briefing on COVID-19 - 11 March 2020. 2020.https://www.who.int/director-general/speeches/detail/who-director-general-s-opening-remarks-at-the-media-briefing-on-covid-19---11-march-2020 (accessed 10 Mar 2021).

2 Chen G, Wu D, Guo W, *et al.* Clinical and immunological features of severe and moderate coronavirus disease 2019. *J Clin Invest* 2020;**130**:2620–9. doi:10.1172/JCI137244

3 Zhou F, Yu T, Du R, *et al.* Clinical course and risk factors for mortality of adult inpatients with COVID-19 in Wuhan, China: a retrospective cohort study. *The Lancet* 2020;**395**:1054–62. doi:10.1016/S0140-6736(20)30566-3

4 Zhu L, She Z-G, Cheng X, *et al.* Association of Blood Glucose Control and Outcomes in Patients with COVID-19 and Pre-existing Type 2 Diabetes. *Cell Metab* 2020;**31**:1068-1077.e3. doi:10.1016/j.cmet.2020.04.021

5 McGurnaghan SJ, Weir A, Bishop J, *et al.* Risks of and risk factors for COVID-19 disease in people with diabetes: a cohort study of the total population of Scotland. *Lancet Diabetes Endocrinol* 2021;**9**:82–93. doi:10.1016/S2213-8587(20)30405-8

6 Barron E, Bakhai C, Kar P, *et al.* Associations of type 1 and type 2 diabetes with COVID-19-related mortality in England: a whole-population study. *Lancet Diabetes Endocrinol* 2020;**8**:813–22. doi:10.1016/S2213-8587(20)30272-2

For peer review only

7 Xu L, Mao Y, Chen G. Risk factors for 2019 novel coronavirus disease (COVID-19) patients progressing to critical illness: a systematic review and meta-analysis. *Aging* 2020;**12**:12410–21. doi:10.18632/aging.103383

8 Huang C, Wang Y, Li X, *et al.* Clinical features of patients infected with 2019 novel coronavirus in Wuhan, China. *The Lancet* 2020;**395**:497–506. doi:10.1016/S0140-6736(20)30183-5

9 Kumar A, Arora A, Sharma P, *et al.* Is diabetes mellitus associated with mortality and severity of COVID-19? A meta-analysis. *Diabetes Metab Syndr Clin Res Rev* 2020;**14**:535–45. doi:10.1016/j.dsx.2020.04.044

10 Regjeringen. Omfattende tiltak for å bekjempe koronaviruset. 2020.https://www.regjeringen.no/no/aktuelt/nye-tiltak/id2693327/ (accessed 10 Nov 2020).

11 Blendon RJ, Benson JM, DesRoches CM, *et al.* The Public's Response to Severe Acute Respiratory Syndrome in Toronto and the United States. *Clin Infect Dis* 2004;**38**:925–31. doi:10.1086/382355

12 Brooks SK, Webster RK, Smith LE, *et al.* The psychological impact of quarantine and how to reduce it: rapid review of the evidence. *The Lancet* 2020;**395**:912–20. doi:10.1016/S0140-6736(20)30460-8

13 Yoon M-K, Kim S-Y, Ko H-S, *et al.* System effectiveness of detection, brief intervention and refer to treatment for the people with post-traumatic emotional distress by MERS: a case report of community-based proactive intervention in South Korea. *Int J Ment Health Syst* 2016;**10**:51. doi:10.1186/s13033-016-0083-5

14 Hetkamp M. Sleep disturbances, fear, and generalized anxiety during the COVID-19 shut down phase in Germany: relation to infection rates, deaths, and German stock index DAX. *Sleep Med* 2020;:4.

15 Yu H, Li M, Li Z, *et al.* Coping style, social support and psychological distress in the general Chinese population in the early stages of the COVID-19 epidemic. *BMC Psychiatry* 2020;**20**:426. doi:10.1186/s12888-020-02826-3

16 Fisher A, Roberts A, McKinlay AR, *et al.* The impact of the COVID-19 pandemic on mental health and well-being of people living with a long-term physical health condition: a qualitative study. Public and Global Health 2020. doi:10.1101/2020.12.03.20243246

17 Marcovecchio ML, Chiarelli F. The effects of acute and chronic stress on diabetes control. *Sci Signal* 2012;**5**:pt10. doi:10.1126/scisignal.2003508

18 Ikesu R, Miyawaki A, Sugiyama T, *et al.* Trends in Diabetes Care during the COVID-19 Outbreak in Japan: an Observational Study. *J Gen Intern Med* Published Online First: 19 January 2021. doi:10.1007/s11606-020-06413-w

19 Pal R, Yadav U, Verma A, *et al.* Awareness regarding COVID-19 and problems being faced by young adults with type 1 diabetes mellitus amid nationwide lockdown in India: A qualitative interview study. *Prim Care Diabetes* 2021;**15**:10–5. doi:10.1016/j.pcd.2020.07.001

20 Shi C, Zhu H, Liu J, *et al.* Barriers to Self-Management of Type 2 Diabetes During COVID-19 Medical Isolation: A Qualitative Study. *Diabetes Metab Syndr Obes Targets Ther* 2020;**Volume 13**:3713–25. doi:10.2147/DMSO.S268481

For peer review only

21 Verma A, Rajput R, Verma S, *et al.* Impact of lockdown in COVID 19 on glycemic control in patients with type 1 Diabetes Mellitus. *Diabetes Metab Syndr Clin Res Rev* 2020;**14**:1213–6. doi:10.1016/j.dsx.2020.07.016

22 Barchetta I, Cimini FA, Bertoccini L, *et al.* Effects of work status changes and perceived stress on glycaemic control in individuals with type 1 diabetes during COVID-19 lockdown in Italy. *Diabetes Res Clin Pract* 2020;**170**:108513. doi:10.1016/j.diabres.2020.108513

23 Aragona M, Rodia C, Bertolotto A, *et al.* Type 1 diabetes and COVID-19: The "lockdown effect." *Diabetes Res Clin Pract* 2020;**170**. doi:10.1016/j.diabres.2020.108468

24 Potier L, Hansel B, Larger E, *et al.* Stay-at-Home Orders During the COVID-19 Pandemic, an Opportunity to Improve Glucose Control Through Behavioral Changes in Type 1 Diabetes. *Diabetes Care* 2021;**44**:839–43. doi:10.2337/dc20-2019

25 Fernández E, Cortazar A, Bellido V. Impact of COVID-19 lockdown on glycemic control in patients with type 1 diabetes. *Diabetes Res Clin Pract* 2020;**166**:108348. doi:10.1016/j.diabres.2020.108348

26 Joensen LE, Madsen KP, Holm L, *et al.* Diabetes and COVID-19: psychosocial consequences of the COVID-19 pandemic in people with diabetes in Denmark—what characterizes people with high levels of COVID-19-related worries? *Diabet Med* 2020;**37**:1146–54. doi:10.1111/dme.14319

27 Forde R, Arente L, Ausili D, *et al.* The impact of the COVID-19 pandemic on people with diabetes and diabetes services: A pan-European survey of diabetes specialist nurses undertaken by the Foundation of European Nurses in Diabetes survey consortium. *Diabet Med* Published Online First: 30 December 2020. doi:10.1111/dme.14498

28 Grabowski D, Overgaard M, Meldgaard J, *et al.* Disrupted Self-Management and Adaption to New Diabetes Routines: A Qualitative Study of How People with Diabetes Managed Their Illness during the COVID-19 Lockdown. *Diabetology* 2021;**2**:1–15. doi:10.3390/diabetology2010001

29 Flanagan JC. The critical incident technique. *Psychol Bull* 1954;**51**:327–58. doi:10.1037/h0061470

30 Fridlund B, Henricson, M., Mårtensson J. Critical Incident Technique applied in nursing and healthcare sciences. *SOJ Nurs Health Care* 2017;**3**:1–5. doi:10.15226/2471-6529/3/1/00125

31 Kemppainen JK. The critical incident technique and nursing care quality research. *J Adv Nurs* 2000;**32**:1264–71. doi:10.1046/j.1365-2648.2000.01597.x

32 Magliah SF, Zarif HA, Althubaiti A, *et al.* Managing Type 1 Diabetes among Saudi adults on insulin pump therapy during the COVID-19 lockdown. *Diabetes Metab Syndr Clin Res Rev* 2021;**15**:63–8. doi:10.1016/j.dsx.2020.12.013

33 Barone MTU, Harnik SB, de Luca PV, *et al.* The impact of COVID-19 on people with diabetes in Brazil. *Diabetes Res Clin Pract* 2020;**166**:108304. doi:10.1016/j.diabres.2020.108304

34 Di Dalmazi G, Maltoni G, Bongiorno C, *et al.* Comparison of the effects of lockdown due to COVID-19 on glucose patterns among children, adolescents, and adults with type 1 diabetes: CGM study. *BMJ Open Diabetes Res Care* 2020;**8**:e001664. doi:10.1136/bmjdrc-2020-001664

For peer review only

35 Tornese G, Ceconi V, Monasta L, *et al.* Glycemic Control in Type 1 Diabetes Mellitus During COVID-19 Quarantine and the Role of In-Home Physical Activity. *Diabetes Technol Ther* 2020;**22**:462–7. doi:10.1089/dia.2020.0169

36 Christoforidis A, Kavoura E, Nemtsa A, *et al.* Coronavirus lockdown effect on type 1 diabetes management on children wearing insulin pump equipped with continuous glucose monitoring system. *Diabetes Res Clin Pract* 2020;**166**:108307. doi:10.1016/j.diabres.2020.108307

37 Capaldo B, Annuzzi G, Creanza A, *et al.* Blood Glucose Control During Lockdown for COVID-19: CGM Metrics in Italian Adults With Type 1 Diabetes. *Diabetes Care* 2020;**43**:e88–9. doi:10.2337/dc20-1127

38 Maddaloni E, Coraggio L, Pieralice S, *et al.* Effects of COVID-19 Lockdown on Glucose Control: Continuous Glucose Monitoring Data From People With Diabetes on Intensive Insulin Therapy. *Diabetes Care* 2020;**43**:e86–7. doi:10.2337/dc20-0954

39 Fisher L, Hessler D, Polonsky W, *et al.* Diabetes distress in adults with type 1 diabetes: Prevalence, incidence and change over time. *J Diabetes Complications* 2016;**30**:1123–8. doi:10.1016/j.jdiacomp.2016.03.032

40 Freeston M, Tiplady A, Mawn L, *et al.* Towards a model of uncertainty distress in the context of Coronavirus (COVID-19). *Cogn Behav Ther* 2020;**13**:e31. doi:10.1017/S1754470X2000029X

41 Lindstrom B. Contextualizing salutogenesis and Antonovsky in public health development. *Health Promot Int* 2006;**21**:238–44. doi:10.1093/heapro/dal016

42 Antonovsky A, Sjøbu A. *Helsens mysterium: den salutogene modellen*. Oslo: : Gyldendal akademisk 2013.

43 Bauer GF, Eriksson M, Espnes GA, *et al.*, editors. *The Handbook of Salutogenesis*. 1st ed. 2017. Cham: : Springer International Publishing : Imprint: Springer 2017. doi:10.1007/978-3-319-04600-6

44 Raknes G, Strøm MS, Sulo G, *et al.* Lockdown and non-COVID-19 deaths: Cause-specific mortality during the first wave of the 2020 pandemic in Norway. A population-based register study. Epidemiology 2021. doi:10.1101/2021.02.09.21251326

45 Polit DF, Beck CT. *Nursing research: generating and assessing evidence for nursing practice*. Tenth edition. Philadelphia: : Wolters Kluwer Health 2017.

46 Malterud K. Qualitative research: standards, challenges, and guidelines. *The Lancet* 2001;**358**:483–8. doi:10.1016/S0140-6736(01)05627-6

47 Knox S, Burkard AW. Qualitative research interviews. *Psychother Res* 2009;**19**:566–75. doi:10.1080/10503300802702105

48 Malterud K. *Kvalitative forskningsmetoder for medisin og helsefag: en innføring*. Oslo: : Universitetsforlaget 2017.

49 Weekly epidemiological update on COVID-19 - 8 June 2021. https://www.who.int/publications/m/item/weekly-epidemiological-update-on-covid-19---8-june-2021 (accessed 12 Jun 2021).

For peer review only

50 Norwegian Institute of Public Health, (NIPH). Statistics coronavirus and covid-19 - Key figures for Norway. 2021.https://www.fhi.no/sv/smittsomme-sykdommer/corona/dags--og-ukerapporter/dags--og-ukerapporter-om-koronavirus/ (accessed 28 Apr 2021).

For peer review only

Table 1 Description of the participants of the study and characteristics of the interview situation.

Person	*Age range*	*DM Duration years*	*A1c mmol/mol*	*Diabetes complications*	*COVID-19*[1]	*Occupation exposed*	*Living alone*	*Interview form*	*Interview duration minutes*	*Site*[2]
1	60s	46	61	yes			no	meeting	56	1
2	40s	23	64	yes			no	video	25	1
3	30s	23	51	yes			yes	meeting	51	2
4	50s	45	66	yes		Kindergarten	yes	video	39	2
5	20s	17	60	no			no	video	17	2
6	40s	3	63	no	(+)		no	video	33	2
7	50s	19	54	no			no	video	59	1
8	20s	25	50	no			no	video	43	3
9	50s	21	51	no	+		no	video	38	3
10	50s	14	66	no	+		no	video	42	3
11	50s	9	63	no			no	video	22	3
12	40s	29	53	yes		School assistant	no	video	42	3
13	30s	20	65	no			yes	telephone	57	3
14	20s	10	46	no		Healthcare	yes	video	27	3
15	50s	53	57	yes			no	video	35	2
16	40s	44	78	no			no	telephone	45	2
17	40s	37	53	no		Teacher	no	video	36	2
18	50s	32	48	yes			no	telephone	93	2
19	50s	26	53	no			yes	video	51	3
Mean	**45 years**	**26 years**	**58 mmol/mol**						**43 min**	

[1] The symbol + denotes a confirmed case of COVID-19 infection, and the symbol (+) denotes suspected but unconfirmed infection.

[2] The three different sites of the study are represented by numbers 1, 2 and 3. The total patient populations at the three hospitals were 160 000 (1), 240 000 (2) and 560 000 (3). According to the annual report from the Norwegian Diabetes Register for Adults, 393 (1), 699 (2) and 1230 (3) patients with type 1 diabetes are treated and followed up at these outpatient clinics.

For peer review only - http://bmjopen.bmj.com/site/about/guidelines.xhtml

Table 2 Interview guide for the study, including probing questions and background information.

TOPIC	Q1	Q2	Q3	Q4	Q5	Q6	**Probing questions**	**Background information**
**Topic1: COVID-19 and daily life and coping with the situation**	When you first heard about the COVID-19 virus and that it had spread to Norway, what were your thoughts?	Do you have any thoughts about whether this virus is dangerous for you, and if so, how have these thoughts affected you?	Have you taken any specific actions to take care of your diabetes treatment during this period?	Can you describe how your everyday life is or was affected by the COVID-19 pandemic?	Can you describe what it was like for you and your family when society started reopening?	Can you describe what it has been like for you to take care of the diabetes treatment during this time?	Where did this happen?<br><br>What did you do in this situation / can you explain more about what happened?<br><br>What did you think in this situation?<br><br>What feelings did you have in this situation?<br><br>What do you think was the most demanding part of this situation?<br><br>What does this mean for you in the future?	For how long have you had diabetes?<br><br>Have you developed any diabetes complications?<br><br>What was your last registered A1c?
**Topic 2: Sources of information about COVID-19 and diabetes**	How did you and your family relate to news and media coverage of the pandemic?	Can you describe how you got information about the COVID-19 pandemic?						
**Topic 3: COVID-19 and help from the healthcare service**	What expectations have you had about possible help / support from the health service to cope with the situation?	Do you feel that your expectations have been met?	If there was a new pandemic, what kind of health service would you wish to be established for people in risk groups?					

For peer review only

Table 3 Data analysis of experiences from quotations, grouped by subcategory, category and main area.

QUOTATION	SUBCATEGORY	CATEGORY	MAIN AREA
"Because it was so uncertain whether I was more at risk or, uhm yes, or how things were because no one knew that much. So, I thought it was scary." (3)	Uncertainty caused by belonging to a high-risk group	Concerns related to COVID-19 and T1DM	Increased psychosocial burden of T1DM during the COVID-19 pandemic
"But I was genuinely afraid of getting this, because I thought that if I get it, I'll die." (9)	Anxiety concerning infection		
"The worst thing about the corona, when it came, it was all the uncertainty – we knew nothing." (18)	Concerns about the overall situation		
"I paid attention at first, and then I thought this is not good, I will get depressed from seeing this, all this intimidating propaganda." (19)	Continuous media focus raised concerns	A need for knowledge about T1DM and COVID-19	
"If I've been missing anything to some extent, it might getting insight into why diabetics are particularly vulnerable in a corona situation." (10)	Need for balanced information for people in risk groups		
"I contacted the doctor's office. I also contacted the hospital to find out if I was at risk and what to do if called back to work." (4)	A desire for personalized information		
"No, it's more the social discomfort you feel when people you meet sort of go for a hug and then you have to say 'You know what? I don't do that'." (14)	Challenges in social distancing	Consequences in social relations from COVID-19-related restrictions	
"And it was – especially for the children – it was probably the most demanding, that they had to avoid being with others for such a long time." (10)	Increased restrictions for family members of people with T1DM		
"So, it was kind of that I should not return to work, so that they [her colleagues] would not get a bad conscience if I were to die." (4)	Restrictions related to being in a high-risk group due to T1DM		
"I felt, when we locked down, that ... that the start was completely awful because it, uhm, it completely changed my routines." (3)	Difficulties creating routines in everyday life at home	Altered routines due to COVID-19 affected T1DM self-management	Changed conditions for T1DM treatment during the COVID-19 pandemic

For peer review only

"Uhm yes, but it makes one stay indoors more and such, and then it's easier to make a little extra-good food so, …" (2)	Difficulties maintaining a healthy lifestyle	
"Surprisingly, the treatment has been better than ever before. But it has to do with the fact that I got a new type of insulin pump. And it has really made everyday life a lot easier." (8)	The benefit of glucose sensor and insulin pump in diabetes treatment	
"I had an incredibly high fever and had an insanely high blood glucose." (9)	Challenging fluctuations in blood glucose during COVID-19 disease	
"It [the blood glucose] has been affected because I, well it's much busier at work [teacher] because we wash desks and surfaces all the time." (17)	New routines at work to prevent the spread of COVID-19	Changes in the work or study situation due to COVID-19 affecting T1DM self-management
"But clearly, the lack of breaks and not being able to eat and such, I must say that I have sometimes felt that it has been tough at work." (17)	Increased workload due to COVID-19 caused stress	
"All the time I feel that I have kind of a 'sting' in the back of my mind, whether I might be laid off again." (18)	Financial uncertainty caused stress	
"It's even easier, when I work from home, to maintain good blood glucose control, compared with running around taking those measurements at work all the time." (10)	More predictable everyday life gave more time to focus on T1DM self-management	
"The one [health check] before the summer was just completely eliminated, so there was a year in between check-ups and I don't think that is good." (13)	Lack of or delayed follow-up from the specialist health service	Reorganization of the health service due to COVID-19 affecting T1DM treatment
"I must admit that in the beginning I thought 'This is a state of emergency, now I have to stock up on insulin'." (15)	Concerns regarding access to medicines	
"And if I should end up with someone who does not necessarily have full control over what blood glucose is all about in such an intensive ward, then yes. (7)	Concerns about ICU healthcare professionals' knowledge of specialised diabetes treatment in case of severe illness and need for help	
"In the end I called the diabetes nurse and asked: 'What am I going to do? I can't get my blood glucose down'." (9)	Availability of healthcare professionals by telephone	

For peer review only

Table 4 Data analysis of actions from quotations, to subcategories, via categories up to the main areas.

QUOTATION	SUBCATEGORY	CATEGORY	MAIN AREA
"I was very, very conscious about keeping my distance to others, and as I said, I wore a mask as well." (18)	Increased awareness of complying with infection control advice	Practical strategies to avoid being infected with COVID-19	Actions to handle psychosocial strain related to T1DM and COVID-19
"Every Saturday he [her brother] goes to the store and buys groceries for me." (15)	Getting help from others to obtain food and medicine		
"We can't let you [the children] take the bus unnecessarily, you should not go to a party where there are 50 people, that is not what we should do now. So yes, they must consider me." (16)	Limiting family members' number of social contacts		
"I have a good friend who has bone marrow cancer. And we both agreed to it because she is very restrictive about who she socializes with too, so the two of us had a lot of contact." (4)	Choosing a few people to meet and socialize with	Measures to maintain social contact with others	
"And in this hobby community they are good at organizing such online meetings, so we sit at our PCs together instead of alone." (12)	Creating alternative arenas for socialization		
"I'm so fortunate that I have a husband that I live with. I'm married, you know, so I think that the loneliness that many feel. I'm much better off." (15)	Finding joy and support within the family		
"And then I thought well, it's not certain that it's really bad that I get it [COVID-19] because I'm pretty healthy, I have a good Hba1c, my immune system is kind of good." (5)	Positive cognitive reflections to deal with the situation	Mental strategies to deal with the situation	
"I kind of tried to avoid maybe the newspapers as much as possible, because I feel that … well, it affects me in a negative sense." (3)	Selective attitude towards media information		
"I asked my doctor what should I do? Should I just continue working from home and answer technical questions, or should I travel and work as normal?" (19)	Actively searching for personalized information		

"I quickly decided that I must live as normally as possible. Eat as normal. Be physically active outside. Keep in touch with others in a safe way." (4)	Creating predictable everyday routines for T1DM self-management	Creating a safe structure in daily life	Actions to handle changed conditions for T1DM treatment during the COVID-19 pandemic
"So then I eat in the car- instead of going in there to eat with the others [on a construction site]." (2)	Starting new routines at work for infection control		
"I've been pretending that it's a normal day. I get up at regular times, shower, put on make-up, etc." (15)	Creating appropriate home-office routines		
"And I have enough things to do so that there will not be much effort spent on thinking too much, to put it that way." (11)	Consciously finding meaningful pursuits to avoid brooding		
"And also I think we are very fortunate to have the diabetes system we have here in Norway; that I was allowed to get Freestyle Libre and such." (10)	Using medical technical equipment actively in treating diabetes	Measures to ensure proper T1DM self-management during the COVID-19 pandemic	
"I could not go to the gym, so I had to use nature instead." (4)	Alternative physical activities to maintain a healthy lifestyle during the COVID-19 pandemic		
"And tried to inject, took higher and higher insulin doses." (9)	Titrate insulin doses in relation to blood glucose fluctuations during COVID-19 disease		
"Then I was isolated in the bedroom by my wife. I was not allowed to go out." (9)	Isolating due to COVID-19 illness		
"And about medicine and stuff. I have always had a stock of insulin." (4)	Keeping a stockpile of insulin available for emergencies		

For peer review only - http://bmjopen.bmj.com/site/about/guidelines.xhtml

COREQ (COnsolidated criteria for REporting Qualitative research) Checklist

A checklist of items that should be included in reports of qualitative research. You must report the page number in your manuscript where you consider each of the items listed in this checklist. If you have not included this information, either revise your manuscript accordingly before submitting or note N/A.

Topic	Item No.	Guide Questions/Description	Reported on Page No.
**Domain 1: Research team and reflexivity**			
Personal characteristics			
Interviewer/facilitator	1	Which author/s conducted the interview or focus group?	
Credentials	2	What were the researcher's credentials? E.g. PhD, MD	
Occupation	3	What was their occupation at the time of the study?	
Gender	4	Was the researcher male or female?	
Experience and training	5	What experience or training did the researcher have?	
Relationship with participants			
Relationship established	6	Was a relationship established prior to study commencement?	
Participant knowledge of the interviewer	7	What did the participants know about the researcher? e.g. personal goals, reasons for doing the research	
Interviewer characteristics	8	What characteristics were reported about the inter viewer/facilitator? e.g. Bias, assumptions, reasons and interests in the research topic	
**Domain 2: Study design**			
Theoretical framework			
Methodological orientation and Theory	9	What methodological orientation was stated to underpin the study? e.g. grounded theory, discourse analysis, ethnography, phenomenology, content analysis	
Participant selection			
Sampling	10	How were participants selected? e.g. purposive, convenience, consecutive, snowball	
Method of approach	11	How were participants approached? e.g. face-to-face, telephone, mail, email	
Sample size	12	How many participants were in the study?	
Non-participation	13	How many people refused to participate or dropped out? Reasons?	
Setting			
Setting of data collection	14	Where was the data collected? e.g. home, clinic, workplace	
Presence of non-participants	15	Was anyone else present besides the participants and researchers?	
Description of sample	16	What are the important characteristics of the sample? e.g. demographic data, date	
Data collection			
Interview guide	17	Were questions, prompts, guides provided by the authors? Was it pilot tested?	
Repeat interviews	18	Were repeat inter views carried out? If yes, how many?	
Audio/visual recording	19	Did the research use audio or visual recording to collect the data?	
Field notes	20	Were field notes made during and/or after the inter view or focus group?	
Duration	21	What was the duration of the inter views or focus group?	
Data saturation	22	Was data saturation discussed?	
Transcripts returned	23	Were transcripts returned to participants for comment and/or	

For peer review only

Topic	Item No.	Guide Questions/Description	Reported on Page No.
		correction?	
**Domain 3: analysis and findings**			
Data analysis			
Number of data coders	24	How many data coders coded the data?	
Description of the coding tree	25	Did authors provide a description of the coding tree?	
Derivation of themes	26	Were themes identified in advance or derived from the data?	
Software	27	What software, if applicable, was used to manage the data?	
Participant checking	28	Did participants provide feedback on the findings?	
Reporting			
Quotations presented	29	Were participant quotations presented to illustrate the themes/findings? Was each quotation identified? e.g. participant number	
Data and findings consistent	30	Was there consistency between the data presented and the findings?	
Clarity of major themes	31	Were major themes clearly presented in the findings?	
Clarity of minor themes	32	Is there a description of diverse cases or discussion of minor themes?	

Developed from: Tong A, Sainsbury P, Craig J. Consolidated criteria for reporting qualitative research (COREQ): a 32-item checklist for interviews and focus groups. *International Journal for Quality in Health Care*. 2007. Volume 19, Number 6: pp. 349 – 357

Once you have completed this checklist, please save a copy and upload it as part of your submission. DO NOT include this checklist as part of the main manuscript document. It must be uploaded as a separate file.

For peer review only
